# The Clonal Diversity of Peripheral B Cell Receptor Immune Repertoire Impaired by Residual Malignant B Cells Predicts Treatment Efficacy in B Cell Lymphoma Patients

**DOI:** 10.3390/cancers14194628

**Published:** 2022-09-23

**Authors:** Meng Wu, Jing Zhang, Yi Wang, Lan Mi, Xiaopei Wang, Weiping Liu, Jie Fu, Haifeng Song, Yuqin Song, Jun Zhu

**Affiliations:** 1 Key Laboratory of Carcinogenesis and Translational Research (Ministry of Education/Beijing), Department of Lymphoma, Peking University Cancer Hospital & Institute, Beijing 100142, China; 2 State Key Laboratory of Proteomics, Beijing Proteome Research Center, National Center for Protein Sciences (Beijing), Beijing Institute of Lifeomics, Beijing 102206, China

**Keywords:** germinal center, B cell receptor immune repertoire, B cell lymphoma, clonal diversity, somatic hypermutation

## Abstract

**Simple Summary:**

This study, for the first time, provided both in-vitro and clinical evidence that established a connection between the presence of residual malignant B cells and the clonal diversity of the resulting BCR repertoire. Lymphoma cells cultured with human germinal center B cells suppressed the normal increase of diversity. In a special setting of stem cell transplant, the retarded growth rate of a patient’s peripheral B cell diversity is of great prognostic potential for future relapse.

**Abstract:**

Germinal center (GC) is the vital locus for the evolution of naïve B cells into memory B and plasma cells, but also a hotbed for the proliferation of malignant B cells. We hypothesized that malignant B cells may locally or globally impact GCs to produce peripheral B cell receptor immune repertoire (BCR IR) with reduced clonal diversity. In this study, we first validated our hypothesis in a novel human in-vitro GC (hiGC) model. The addition of the diffuse large B cell lymphoma (DLBCL) cells to the hiGC culture attenuated the rate of diversity growth. For clinical validation, we collected samples from 17 DLBCL patients at various points during high-dose therapy and autologous stem cell rescue. The elimination and reestablishment of the patients’ lymphatic pool allowed us to unambiguously monitor the impact of tumor cells on the replenishment of the peripheral BCR IR. Compared to the nine patients who did not relapse after treatment, relapsed patients tended to have a slower rate of recovery regarding the clonal diversity of their peripheral BCR IR. Our results suggest a mechanistic and clinical connection between residual tumor cells and abnormal peripheral BCR IR, which may corelate with treatment efficacy in B cell lymphomas.

## 1. Introduction

B cell lymphomas constitute the majority (>80%) of the non-Hodgkin lymphomas (NHL) and are a diverse collection of lymphoproliferative disorders originating in various stages of B lymphocytes. Germinal centers (GC) within the B cell follicles in the secondary lymph nodes are considered the hotbeds for the oncogenic transformation of B cells due to their high mutational and cell proliferation rates that are indispensable for the neoantigen formation and affinity maturation of naïve B cells [1,2,3]. The relationship between affinity maturation and GC apoptosis is complex [4]. Notably, any kind of pathogenic contribution of the genetic and/or environmental etiological factors modulating the normal physiological processes of GC can act as the fundamental mechanism to induce NHL pathogenesis, however, with only two rare exceptions of lymphoblastic and mantle-cell lymphomas [5]. Pathologically not all types of B cell lymphomas initiate at the GC, but most of the NHL subtypes exploit the high proliferation capacity of GC-forming B cells for their clonal expansions [6,7]. Interestingly, when GC constituting normal B cell population undergoes rapid apoptosis due to inadequate ligation of B cell receptors (BCR) in the light zone (LZ), neoplastic B cells can efficiently evade the GC’s selection mechanism to survive from apoptosis, and thus, continue to proliferate uncontrollably to produce the tumor mass [8,9,10]. In addition, normal GC B cells accumulate elevated levels of somatic hypermutation (SHM) for the diversification of BCR immune repertoire (BCR IR), while tumor B cells often proliferate with limited alteration of BCR sequence despite the expression of activation-induced cytidine deaminase (AICDA) [11,12]. Therefore, these findings suggest that tumor B cells may locally or globally impact GCs to produce BCR IR with reduced clonal diversity, in contrast to the GCs in healthy individuals.

In this study, we adopted a two-pronged approach to validate our hypothesis by exploiting (1) an in-vitro model of human germinal center (hiGC) containing both normal and malignant B cells and (2) a clinical model involving diffuse large B cell lymphoma (DLBCL) patients who underwent high-dose therapy and autologous stem cell rescue (HDT-ASCR) treatment. We followed the methodological frameworks of previously generated in-vitro human lymphoma and in-vivo murine GC models to establish our in-vitro GC model [13,14,15,16,17,18,19,20,21]. By optimizing the population of seeding tumor cells in the hiGC model, we successfully reproduced the in-vivo effect of malignant B cells in a semi-quantitative manner. Furthermore, BCR IR analysis and transcriptomic profiling by next-generation sequencing (NGS) allowed us to monitor the time-resolved evolution of BCR IR diversity and a wide variety of critical hallmarks of GC forming B cells [22,23,24,25,26]. In the clinical model of HDT-ASCR therapy, the patients’ lymphocytic pool was first eliminated by high-dose chemotherapy, followed by a replenishment after the transplantation of hematopoietic stem cells (HSCs), which allowed us to monitor BCR IR repopulation and diversification while minimizing the interference from existing BCR clones. However, the resulting peripheral BCR IR could be highly sensitive to the negative impact of malignant B cells. Given that the monoclonal proliferation of tumor B cells would compromise the repertoire of activated B cells entering the circulation, we hypothesized that the impact of malignant B cells might be imprinted in the clonal diversity of the BCR IR in the post-ASCR therapy. Hence, patients with residual tumor B cells after HDT-ASCR therapy may present peripheral BCR IR with limited diversity, and eventually relapse, while those who do not relapse may possess a highly diversified peripheral BCR IR population. 

Thus, our results showed that the presence of residual DLBCL cells could significantly impair the normal physiological diversification of repertoire B cells. To measure the peripheral BCR clonal diversity, we employed the Shannon diversity index (SDI), as a holistic measure to identify both in-vitro and in-vivo impacts of malignant B cells on the B cell repertoire. In addition, our findings suggest that the pathological linkage between the tumor B cell population and limited diversification of the peripheral BCR IR population may be utilized as a prognostic indicator for the presence of residual tumor cells in the host system.

## 2. Materials and Methods

### 2.1. Cells Lines and Cell Culture

The MRC-5 cell line was purchased from American Type Culture Collection (ATCC, Rockville, MD). The Karpas422 cell line was a gift from Dr. Kai Fu at the University of Nebraska, and the WSU-NHL cell line was a gift from Dr. Zhengying Pan at Peking University. All cells were cultured in RPMI1640 medium supplemented with 10% heat-inactivated fetal bovine serum (FBS) (Gibco, NYC, NY, USA). Human PBMC cells were purchased from Sailybio (Sailybio Tech Co., Ltd., Shanghai, China). NB cells were isolated from PBMC using the naïve B isolation kit (Miltenyi, Bergisch Gladbach, Germany), according to the manufacturer’s protocol.

### 2.2. Lentiviral Transduction of Cell Lines

Lentivirus particles carrying the coding sequence of CD40L, BAFF, and EGFP (lenti-CD40L-BAFF) were purchased from OBIO Technology (OBiO Tech Co., Ltd., Shanghai, China). A detailed protocol of constructing the vector can be found in the Supplementary Materials. MRC-5 cells (1 × 10^5^) were seeded onto a 6-well plate 24 h prior to the addition of the lenti-CD40L-BAFF virions at a multiplicity of infection (MOI) of 30. Then cells were allowed to grow for 72 h in a fresh medium before selecting with puromycin (Thermo, Long Beach, CA, USA) at a final concentration of 1 μg/mL for the isolation of stably transfected cell lines. Lenti-mCherry virus particles were purchased from OBiO (OBiO Tech Co., Ltd., Shanghai, China), which was used to transduce Karpas422 and WSU-NHL (1 × 10^5^ cells/mL) cells. Stably transfected DLBCL cells were selected by adding puromycin (1 μg/mL).

### 2.3. In Vitro Culture of Human GC B Cells with and without Tumor Cells

The hiGC B cells were cultured based on a protocol with slight modifications [17,18]. Briefly, in a 6-well plate previously seeded with MRC40LB (2 × 10^5^ cells per well) cells, freshly isolated NB cells (2 × 10^5^ cells per well) were co-cultured in a BCM medium containing hIL-4 (2 ng/mL, day 0). On day 4, the hiGC B cells were re-plated onto a freshly seeded feeder layer and cultured for another 15 days in the previous medium supplemented with 10 ng/mL hIL-21 (Sinobiological, Beijing, China). For the co-culture of abnormal hiGC model containing tumor cells, varying numbers of mCherry+ Karpas422 or WSU-NHL cells were mixed with NB cells before being seeded onto the MRC40LB feeder layer. The co-cultured cells were maintained in the same medium as the normal hiGC culture.

### 2.4. Flow Cytometry (FCM) Analysis

FCM was carried out on a NovoSampler Pro or a NovoSampler Q (NovoCyte, Surrey, UK) system and data were analyzed using the NovoExpress Software 1.4.1 (NovoCyte, Surrey, UK). A detailed description of the sample preparation procedure and purchasing information of the antibodies used in the FCM assays can be found in the Supplementary Materials.

### 2.5. RNA-Seq Analysis

For RNA seq analysis, total RNA was extracted from hiGC cells on day 0, 8, 12, 14, and 19, respectively, and were sent to Novogene (Novogene Co., Ltd., Beijing, China) for RNA-seq analysis. A detailed description of the RNA-seq procedure can be found in the Supplementary Materials.

### 2.6. Patients’ Characteristics and Clinical Samples Collection Procedure

Peripheral blood samples were collected from 17 patients who achieved CR before receiving HDT-ASCR therapy (including 3 cases with consolidative transplantation after first-line therapy and 14 cases with salvage transplantation after salvage therapy), as well as 12 healthy volunteers as controls. All the patients had a pathological diagnosis of DLBCL, according to the 2016 WHO criteria [27]. Response criteria were defined according to the Lugano 2014 guidelines [28]. We collected samples from each patient at least at three time points (TP): (1) before the administration of the conditioning regimen (TP1), (2) immediately before stem cell transfusion (TP2), and (3) 6–8 weeks after HDT-ASCR therapy (TP3), while for those who relapsed we collected samples from the fourth time point of confirmed relapse (TP4). The study protocol was approved by the Medical Ethics Committee of Peking University Cancer Hospital & Institute and the necessity for patient informed consent was waived (No. 2020KT93).

### 2.7. BCR IR Analysis

RNA extracted from patient-derived blood samples or hiGC cultures were analyzed for integrity and size distribution on an Agilent 2100 Bioanalyzer with the Agilent RNA 6000 Pico Kit (Agilent Technologies, CA, USA). Samples were then reverse transcribed using complementary DNA (cDNA) synthesis kit, from which the BCR CDR3 was amplified by multiplex PCR. A total of 267 forward primers for the V regions (IgH, IgK and IgL) and 26 reverse primers for the junction (J) regions were used (GS Medical, Beijing, China). The targeted amplicons (about 100~300 bp) were then purified with VAHTS DNA Clean Beads (Vazymebiotech, Nanjing, China) and processed for quality control with an Agilent 2100 Bioanalyzer before subjecting to the sequencing reaction. RNA barcoding and library preparation were prepared using the Illumina protocols and sequenced using 150 bp paired-ended reads on an Illumina Novaseq 6000 platform (Illumina, San Diego, CA, USA), from which the results were processed using MiXCR (http://mixcr.milaboratory.com, accessed on 20 January 2021). As quality control, the dataset of the mixed hiGC culture was processed with the TRUST4 algorithm [29], from which the IgKappa SDI data were calculated and compared with the original data. A detailed description of the BCR IR sequencing procedure can be found in the Supplementary Materials.

The network generation algorithm and calculation of network properties have been described previously [23,30]. Briefly, each vertex represented a unique sequence, and the relative vertex size was proportional to the number of identical reads. Edges joined vertices that differed by single nucleotide differences. Clusters were collections of related, connected vertices. A clone (cluster) referred to a group of clonally related B cells, each containing BCRs with identical CDR3 regions or differing by single point mutations. Diversity measures were calculated based on previously reported formulae [31,32], and normalized to the corresponding mean indices of healthy volunteers. The detailed procedure can be found in the Supplementary Materials. ROC and statistical analyses were carried out using R with ‘pROC’ [33] and ‘rstatix’ [34] packages.

## 3. Results

### 3.1. Establishment of a Human In Vitro hiGC Model

**Figure 1:**
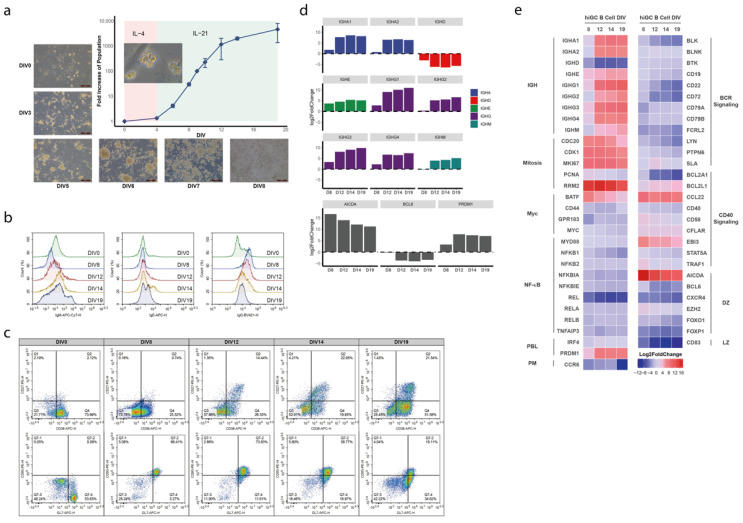
Phenotypic and molecular characterizations of the hiGC culture. (**a**) Growth curve and microscopic images of human in-vitro germinal center (hiGC) B cells co-cultured on the MRC40LB feeder layer. In the first phase of culture (days in vitro (DIV) 0–4), only IL-4 was added; the second phase of culture (DIV4-19) included additional IL-21 in the medium. The insert image in the growth curve plot is a 40× magnification of hiGC B cells exhibiting typical morphological changes upon activation. (**b**) Flow cytometry (FCM) plots showing the increase of IgM+, IgE+, and IgG+ cell populations. (**c**) FCM results showing the increase of CD27+/CD38+ and Fas+/GL7+ cell populations. (**d**) Heatmap of transcription levels for key B cell genes as a function of DIV in the hiGC co-culture. All transcription levels were normalized to those of the primary naïve B cells on DIV 0. PM = precursor of memory cell, PBL = plasmablast, DZ = dark zone, LZ = light zone. (**e**) Detailed trends of transcription levels for all Immunoglobulin heavy chain (IGH) isotypes and critical germinal center genes (AICDA, BCL6, and PRDM1).

To establish the hiGC model, we engineered the human lung fibroblast MRC-5 cell line to stably co-express membrane-bound CD40 ligand (CD40L) and B-cell activating factor (BAFF) as the feeder cell line to promote optimal proliferation of B cells (Figure S1). The resulting cell line, called MRC40LB, exhibited improved growth of human naïve B (NB) cells compared to mouse NIH3T3 feeder cells. Human NB cells were isolated in high purity by the magnetic activated cell sorting from human peripheral blood monocytes (PBMC) based on the expressions of cell surface biomarkers, like CD19+/CD27−. Under the stimulations of CD40L, BAFF, and IL-4, NB cells showed a clear pattern of two-phase exponential growth (Figure 1a). We observed that the seeded NB cells were efficiently attached and clustered to form colonies onto the feeder layer before the manifestation of characteristic morphological changes indicating B cell activation (Figure 1a). Cell proliferation was prominent upon the addition of IL-21 to the medium after 4 days of in-vitro culture. Notably, the NB cell population was rapidly expanded starting from 2 × 10^5^ cells to at least 2000 folds more on the 14th day of culture, and then the growth rate was significantly slowed down. Next, we collected a sizable population of IgG/M/E+ cells primarily constituting the GC B cell population on day 12 when cells were transitioned from SHM to the class switch recombination (CSR) status (Figure 1b), a typical trajectory of GC maturation. We further confirmed the B-cell phenotypes in terms of increased expressions of CD27, CD38, CD95, and GL7 factors by flow cytometry (Figure 1c). Notably, CD27 expression began to emerge on day 8, then reached and maintained the maximum level from day 12 to 19. Next, we investigated the time course of B-cell type marker expressions in the hiGC model B cells by RNA sequencing (RNA-seq) analysis. Transcriptional levels important biomarkers previously identified in primary GC B cells derived from human tonsil [35,36,37] were highlighted in Figure 1d,e. Our results showed that hiGC B cells expressed and maintained AICDA (AID) at a 216-fold higher level than unstimulated NB cells on day 0 throughout the culture period (Figure 1d). Interestingly, mitotic proliferation-associated genes were highly up-regulated from the day 8 to 19, while genes of the BCR signaling pathway and LZ signatures stayed roughly unchanged compared to day 0.

**Figure 2:**
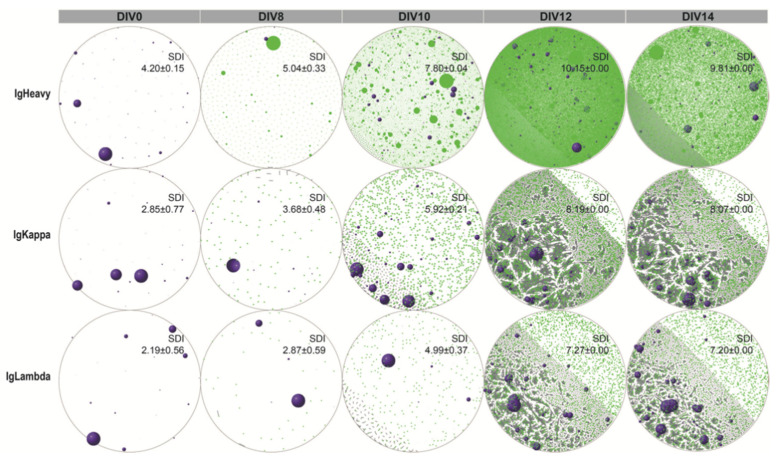
Network plots of human in-vitro hiGC B cells. Purple spheres denote B cell receptor (BCR) clones detected in the original naïve B cell repertoire. Green circles/dots correspond to newly emerged clones. Gray lines connect vertices differing by one nucleotide.

Our study revealed that day 12 appeared to be the critical time point as the trend from day 8 to day 12 underwent sudden alteration. The expression of PR/SET Domain 1 (PRMD1), a key indicator of plasma cell maturation, was found to peak on day 12, concurrent with the transcription of immunoglobulin heavy-chain genes (*IgHA*/*E*/*G*/*M*) (Figure 1d). Moreover, the expression of B-cell lymphoma 6 (BCL6) showed marked reduction on day 12, corroborating with flow cytometry (FCM) results and consistent with the observation that the hiGC B cells could transition from a dark zone (DZ) phenotype to precursor memory B or plasmablast cells. Notably, BCR clones that could not be detected in the starting NB pool were detected between day 12 and 14 upon a high level of AICDA expression, as shown by the network plots in Figure 2. Furthermore, the clonal diversity of BCR IR kept increasing until reaching the maximum on day 12 as measured by SDI. This feature coincided with the emergence of the *IgHA*/*E*/*G*/*M* gene transcriptions, signifying the self-driven transition of the hiGC B cells from the SHM to the CSR stage.

### 3.2. Co-Culture of NB Cells with GC B-Cell (GCB) Type DLBCL Cells in the hiGC

After successfully establishing the hiGC model culture, we investigated the impact of malignant B cells on normal GC reaction in the co-culture of NB and DLBCL cells. We began with two well-characterized GCB-type DLBCL cell lines, namely WSU-NHL:mCherry (CD40+/BAFF-R+) and Karpas 422 (CD40−/BAFF−R−). Neither Karpas 422, nor WSU-NHL, had any detectable expression of AICD before and during the hiGC culture, suggesting the absence of SHM status. After being seeded onto MRC40LB feeder cells, WSU-NHL remained viable but with minimal growth (Figure S2). While Karpas 422 cells had an intrinsically high proliferation rate, which was also maintained during the co-culture condition in the hiGC model. Hence, this line was selected for subsequent use.

**Figure 3:**
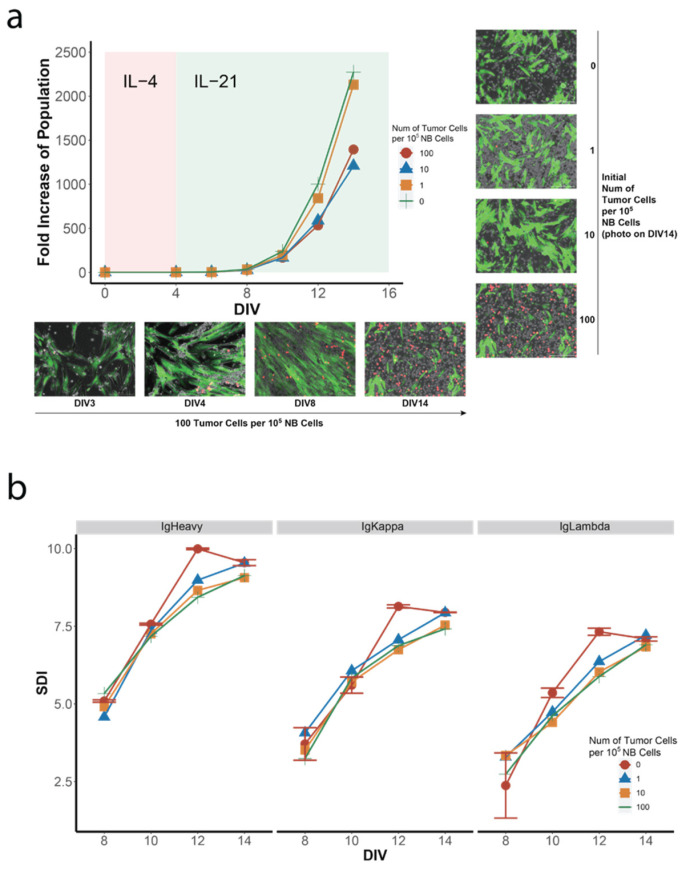
The presence of tumor cells affects both the growth and diversity generation of GC B cells. (**a**) Growth curves and representative microscopic images (merged phase contrast and fluorescence images) of co-cultured human in-vitro hiGC B and Karpas 422 cells. MRC40LB cells exhibited green fluorescence due to stable expression of EGFP; red cells were Karpas 422 cells, stably expressing mCherry, while the normal hiGC B cells were colorless. For the co-culture starting with 100 tumor cells per 10^5^ NB cells, images were taken on DIV3, 4, 8, and 14 (bottom panel). For co-cultures starting with 0, 1, 10, and 100 tumor cells per 10^5^ NB cells, photos were taken on DIV14 (right panel). (**b**) Shannon diversity index (SDI) curves of co-cultured hiGC B and Karpas 422 cells. The reduction in clonal diversity was most prominent on DIV10 and DIV12. Data of pure hiGC B cells were retrieved from two separate cultures, and error bars represented standard deviation (SD).

To develop the co-culture hiGC model, including the Karpas 422 line, we added 1, 10, and 100 Karpas 422 cells to a starting pool of 10^5^ unstimulated NB cells for hiGC expansion, corresponding to 0.001%, 0.01%, and 0.1% relative populations. The cell mixtures exhibited exponential growth, similar to that of a normal hiGC culture (Figure 3a). However, when the co-cultured cells were harvested for BCR IR diversity analysis on day 14 of culture, the proportion of Karpas 422 cells was increased by about 200-fold, as 0.27%, 2.47%, and 17.12% mCherry+ cells were detected by FCM, respectively (Figure S3). Similar to the pure GC culture, the mixed GC and tumor B cells were detached and replated at lower density (10^5^ cells per well) every 4 days after day 8 for replenishment of essential nutrition and room of proliferation. Regarding the mixed GC culture, which had merely half of the population size of the normal GC culture on Day 12 (Figure 1a and Figure 3a), the observed reduction in growth rate of GC B cells was unlikely due to a limit of nutrition or space, but rather an impact from the tumor cells was observed. Since Karpas 422 cells lacked stably expressing BCR gene due to ongoing V (D)J recombination [38], we did not observe a monoclonal expansion of a single predominant BCR clone in the BCR sequencing results (Table S1). Nonetheless, the proliferation of malignant B cells in the hiGC co-culture significantly limited the rate of diversification compared to those of the normal hiGC culture (Figure 3b and Figure S4). Although we observed a slight trend of dose-dependency as the higher population of malignant B cells gave rise to lower BCR IR diversity on day 12, however, the most significant drop in clonal diversity was from zero to 0.001% tumor cells at the same time point. Unlike the hiGC culture containing only normal NB cells, which reached maximal diversity on day 12, hiGC co-culture including tumor B cells could not reach similar diversity levels until day 14, further supporting our hypothesis of suppressed GC clonal diversity growth in the presence of B lymphoma cells.

### 3.3. Comparison of Peripheral Blood BCR IR between DLBCL Patients and Healthy Volunteers

To further verify our hypothesis in a clinical setting, we collected peripheral blood samples from 12 healthy volunteers and 17 DLBCL patients who achieved complete remission (CR) from prior treatment and subsequently received HDT-ASCR regimen. The information of the patients’ demographic background, treatment history, and clinical outcome are listed in Table 1. With a median follow-up time of 28.5 months, 8 out of 17 patients relapsed. Three patients had tumor samples available for BCR IR analysis, and all were of the non-GCB type, designated based on the Hans immunohistochemistry (IHC) algorithm. Notably, two of these patients (#419 and #457) relapsed in 18.8 and 12.6 months after the completion of the HDT-ASCR therapy, respectively, while the third patient (#548) did not relapse until 32.5 months after the completion of treatment based on the last visit in April 2020.

**Figure 4:**
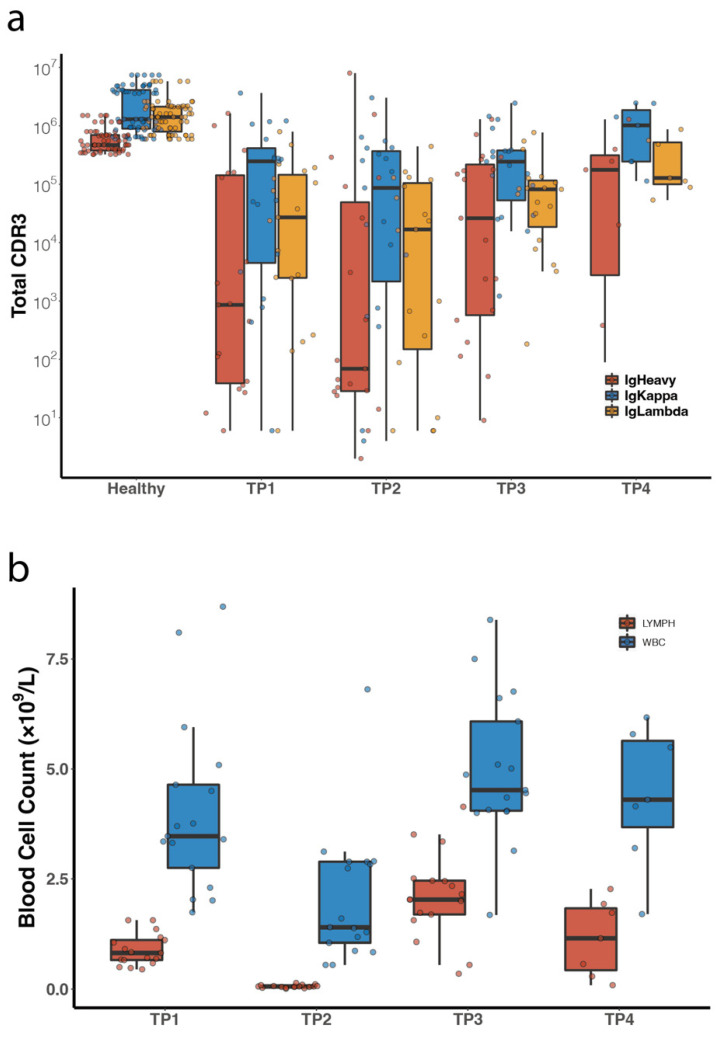
Population measures of the patients’ peripheral blood samples. (**a**) Total CDR3 reads from blood samples of healthy volunteers and DLBCL patients. (**b**) Populations of lymphocytes (LYMPH) and white blood cells (WBC) in patients-derived samples.

The most striking difference between the patient-derived samples and healthy controls was the abundance or overall quantity of BCR clones, even for the TP1 samples which were collected before the commencing of the ASCR treatment. While the total reads of the third complementarity determining region (CDR3) of Igκ from healthy volunteers were 2.77 ± 2.16 × 10^6^ per 4 mL of the blood sample, total Igκ CDR3 reads from the patient-derived samples were at least one order of magnitude lower, except for samples from TP4 when the patient relapsed (1.13 ± 1.01 × 10^6^, Figure 4a). The abundance of patient BCR IR at TP4 indicated that the significantly reduced BCR clonal population between TP1 and TP3 was not due to RNA degradation during cryo-storage, rather due to the state of myeloid suppression throughout the treatment course (Figure 4b). At TP2, in particular, when the patient’s myeloid system was destructed by high-dose chemotherapy, the concentration of intact mRNA from the blood sample was so low, that the mRNA from the entire 4 mL blood sample had to be used for adequate reading of BCR IR clones, in contrast to samples from other time points in which only a small fraction of the mRNA pool would suffice for downstream processing. Consequently, the total counts of BCR clones at TP2 were skewed to comparable levels to other timepoints. VDJ preference analysis showed that there existed several germline V genes that were absent from the pool of healthy volunteers (Figure S5). This was particularly prominent for relapsed patients who had more “abnormal” germline V gene usage than patients who did not relapse.

### 3.4. Characterization of Tumor BCR Clones in Peripheral BCR IR

**Figure 5:**
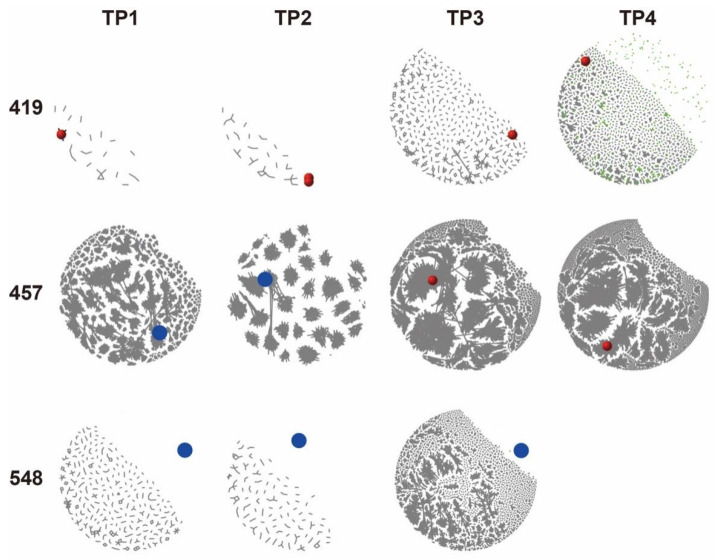
Network plots of the IgKappa (Igκ) CDR3 repertoires from the three patients with available tumor samples. The primary cluster tumor BCR clones were spiked-in, in which red spheres denoted minimally residual disease (MRD) existing in the patients’ blood samples, while blue circles denoted tumor clones not detected in the blood samples. Green circles/dots denoted normal peripheral BCR clones connected by gray lines for evolutionary relation. The layout of nodes on the network plot was based on the Levenshtein distance (LD) between nodes (clones). Patient 548’s peripheral BCR repertoire had no clone that was related to his tumor BCR clone.

Among the 17 patients, we could collect tumor samples from three patients (#419, #457, and #548), which allowed us to identify and quantify the tumor BCR clones in the peripheral BCR IR population. The primary tumor BCR cluster (one cluster of clones collectively accounted for no less than 5% of total tumor BCR population) of these three tumor samples contained κ subtype light chain (Table 2). We then searched for matches between the sequences of the primary tumor BCR cluster and the peripheral BCR IR population at various time points during treatment. For visualization of tumor BCR clones in the network plots, we spiked the Igκ primary tumor BCR cluster into the BCR repertoires from various time points. In the network plots of the combinatorial BCR repertoires, tumor clones were colored red if they pre-existed in blood samples (minimally residual disease, MRD), or were colored blue otherwise (Figure 5). For patient #548, who did not relapse, neither tumor BCR clone nor any other BCR clone phylogenetically related to the tumor clone was detected in samples from any of the three time points. For patients #419 and #457, the red sphere in the network plots indicated that the blood samples contained BCR clones of the primary tumor cluster. Tumor BCR clone was detected in samples from all four time points in the case of patient #419, while patient #457’s tumor BCR clone was only detected at TP3 and TP4. In his/her TP1 and TP2 samples, we found BCR clones whose Levenshtein distance (LD) was 1 (Table 2), suggesting that there might be ongoing clonal evolution in patient #457’s tumor. Indeed, as the layout of vertices in the network plot was based on the Fruchterman–Reingold algorithm (FRA), the tumor BCR clones of patients #419 and #457 were phylogenetically in close proximity to other blood-derived BCR clones, while patient #548’s spiked-in tumor BCR clone was positioned away from other BCR clones (Figure 5). While tumor BCR clones were present in all blood samples of patient #419, they only accounted for 0.18%, 1.4%, 0.2%, and 1.4% of the total peripheral BCR IR at TP1, TP2, TP3, and TP4, respectively. However, for patient #457, the relative population of tumor BCR IR in the total peripheral BCR IR was not more than 0.003% at all time points, albeit the tumor BCR clone was clearly detected at TP3 and TP4.

### 3.5. Diversity Analysis of All Peripheral Blood Samples

**Figure 6:**
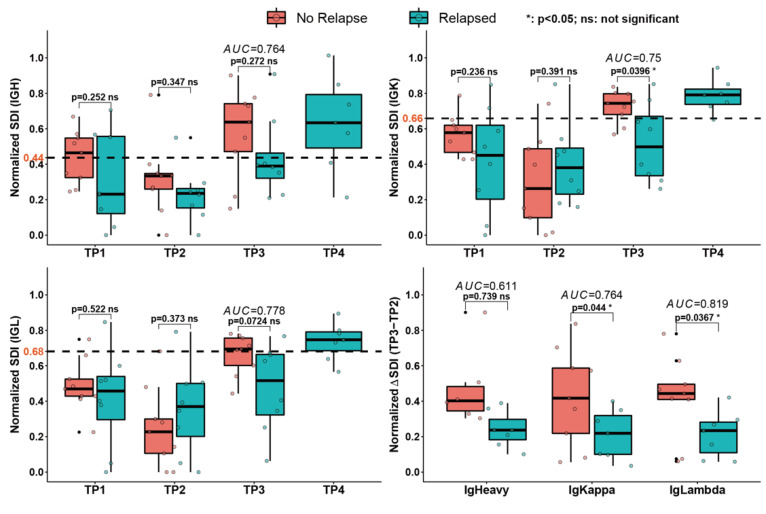
SDIs of peripheral BCR IR from DLBCL patients. The indices were normalized to the mean SDIs of healthy volunteers. T-test and ROC analysis between relapsed and non-relapse groups were carried out using R packages “rstatix” and “pROC”. Dashed horizontal lines marked the calculated cut-off levels (labeled in orange) for the corresponding diversity measures. Area under curve (AUC) values from the ROC analysis were labeled in italic.

We next determined whether the small portion of peripheral BCR IR taken up by tumor BCR clones would affect the overall clonal diversity. As recommended by previous protocols [23,30], we calculated the Gini index, Gini-Simpson Coefficient, and SDI for all peripheral blood samples from patients and healthy volunteers, either with individual BCR clones or clusters as established by network analysis (Table S2, Figure S6). In the ROC analysis, the dichotomous cut-off values of SDI produced a significant (AUC > 0.75) discrepancy between relapsed and non-relapse patients (Figure 6). Especially the ΔSDI and the increase of SDI in the 6–8 weeks period after transplant were significantly different between the relapsed and non-relapsed patients, indicating that HDT therapy led to a significant drop in SDI (TP2), which began to recover after the stem cell transplantation at TP3. The replenishment of the lymphocytic population was susceptible to the influence of tumor cells. Patients who were completely rid of tumor cells tended to have higher SDI at TP3, i.e., faster recovery from the low SDI at TP2 than those who later relapsed. For patients who had residual tumor cells and were susceptible to relapse, their SDI did not reach a similar level until they relapsed at TP4, a trend highly resembling the observations from the hiGC co-culture model.

## 4. Discussion

Through the hiGC co-culture system, we recapitulated the major diversity-generating process in a model of the activated human GC. The NB cells adopted the DZ-state from day 0 to 12 and transitioned to precursor memory B or plasmablast cells between day 12 and 19. Moreover, significantly elevated expression of AICDA warranted widespread SHM and diversification of the CDR3 repertoire. The current hiGC model lacked the Fas/CD95-mediated apoptosis mechanism, which was crucial for the negative selection of low-affinity GC B cells during the affinity maturation process in vivo [8,9]. The absence of the Fas-FasL mechanism in our model allowed unambiguous investigation of the SHM process during GC reaction. Consequently, a large number of hiGC B cells that could have been eliminated in vivo remained proliferating and further contributed to the overall diversity. On the other hand, DLBCL cells possess a defective Fas/CD95 apoptosis pathway conferring resistance to Fas-mediated apoptotic clearance in vivo [39]. Therefore, it can be postulated that in a fully functional GC under the negative influence of malignant B cells, normal B cells would be disproportionately removed due to a lack of affinity for the antigen presented by follicular dendritic cells (fDC) and follicular helper T cell (Tfh), compared to the unrestrained proliferation of tumor B cells. The relative population of tumor B cells in the circulation would be even greater in vivo than what we observed in our hiGC co-culture model lacking the Fas-FasL mechanism. Consequently, the impact of tumor cells on a patient’s BCR repertoire diversity might be more prominent than what we observed in vitro.

The HDT-ASCR therapeutic procedure presents an ideal clinical model for investigating the impact of residual tumor cells on normal GC physiology. Although the peripheral BCR IR accounts for only 2% of the entire BCR IR in a human body, its enormous population size may still easily overwhelm the effect of a few tumor-residing GCs. In the HDT-ASCR scenario, patients’ peripheral blood BCR repertoire at TP1 might be in a suppressed state due to the previous round of treatment. The HDT entailed another serious blow to the overall pool of myeloid and lymphocytic cells and the elimination of all interference from the pre-existing B cell population. After transplantation, the newly established HSC pool restarted to produce a limited number of NB cells, part of which would differentiate into plasma and memory B cells. In addition, patients receiving HDT-ASCR were housed in the same laminar flow ward so that their peripheral B cells could evolve and differentiate under exposure to similar exogenous antigens. Thus, the evolution of the peripheral B cell repertoire from TP2 to TP3 represented a simple and homogeneous process with definitive duration, relative to the evolution of the B cell pool outside the laminar flow ward, where each patient might be exposed to a wide collection of antigens. The GC reaction during the period from TP2 to TP3 was highly sensitive to the presence of DLBCL cells. SDI values from both hiGC co-cultures and clinical samples demonstrated a suppressed rate of diversity growth in the presence of tumor cells.

Based on our analysis of clinical samples, the SDI and ∆SDI exhibited adequate sensitivity to the presence of malignant B cells and possessed great robustness despite the complications during the treatment course, such as systemic infection and dosage reduction due to intolerance. Although the dichotomous cut-off values determined by the ROC analysis might be skewed due to the small number of samples, the discrepancy in the growth of SDI was significant between the relapsed and non-relapse patients and significantly corroborated with the results from the hiGC co-culture model. Limited sampling efficiency is an intrinsic issue of IR analysis with peripheral blood samples, which can only be mitigated but not be fully eliminated [26,40,41]. Based on the results from our tumor samples and previous reports, solely relying on the detection of the circulating tumor clone itself could lead to misguided decision-making [42]. The substantial probability of missing the tumor BCR clone in the blood samples, and the scarcity of tumor samples for extracting tumor BCR sequences when patients were presented for HDT-ASCR, made the holistic approach of SDI measurement a more tractable strategy for determining the existence of malignant B cells.

Our hiGC model thus demonstrated that tumor cells in the hiGC co-culture not only significantly slowed down the rate of clonal diversification but also affected the rate of cell proliferation (Figure 3a). Moreover, increasing the tumor cell population further reduced the overall clonal diversity. The impact from tumor B cells was not due merely to a competition of nutrients and space, as the cell culture was routinely replated to maintain adequate room for growth. Moreover, the population of tumor B cells was too minute to out-compete with normal B cells with respect to proliferation and diversification. Both the in-vitro and clinical models showed that tumor cells with a population as low as 0.1% would suffice to profoundly impair the normal GC physiology. Furthermore, paracrine mechanisms could also be involved in the process of suppressing the growth of normal GC B cells, which remains to be elucidated.

A few caveats existed in this study. Firstly, it remains to be determined whether the impact of Karpas-422 cells on normal GC B cells will be generalizable to other B cell lymphoma cell lines of GC origin. Secondly, the limited availability of tumor samples prevented us from evaluating the correlation between the Levenshtein distance of BCR clones and risk of future relapse. For the case of patient 548 who did not relapse, the tumor BCR clone was remote from all blood BCR clones, whereas patients 419 and 457 had a large number of peripheral BCR clones closely related to the tumor BCR clone before their relapse. Future studies are needed to analyze more tumor samples against corresponding blood samples. Thirdly, we did not collect TP4 blood samples from patients who did not relapse. It thus remained to be seen whether the impairment of tumor cells on diversity would persist beyond TP3. That is, in future studies, it will be desirable to collect TP4 blood samples for patients without relapse and compare their diversity indices with those of the relapsed patients. Most importantly, the current in-vitro hiGC and clinical ASCR models lack the ability to fully discern the molecular mechanisms by which the B lymphoma cells impacted GC reactions. Such mechanisms might involve tumor cells residing in certain GCs and exhibit local impairment on GC reactions, resembling the hiGC model. It may also be possible that the tumor cells conferred systemic suppression of all GCs.

## 5. Conclusions

Collectively, we validated the hypothesis of GC reaction suppression by DLBCL cells in a hiGC co-culture model and correlated our findings to a set of clinical samples from DLBCL patients. Both the in-vitro and clinical studies demonstrated that, it is the dynamic growth of clonal diversity in GC that is most sensitive to the negative impact of tumor B cells. The dynamic trend SDI was an efficient and robust gauge of the overall normalcy of GC reactions, in which a lower than the normal value indicated the presence of residual tumor cells. Clinical trials and in vitro studies in other types of B cell lymphomas are thus warranted to further strengthen the mechanistic and clinical connections among residual tumor cells, suppressed GC reaction, and peripheral BCR IR population.

## Figures and Tables

**Table 1:** Patients’ demographic background, treatment history, and clinical outcomes.

No.	Age	Sex	Subtypes	Status	Conditioning Regimen	Complication during ASCR	Progression	PFS
598	36	M	GCB	Salvage	CBV	Pneumonia	No	35.5
592	51	M	non-GCB	Salvage	BEAC	-	Yes	9.3
602	29	M	non-GCB	Salvage	CBV	-	No	34.0
535	23	F	GCB	Consolidation	CBV	-	Yes	4.9
457	45	M	non-GCB	Salvage	CBV	-	Yes	12.6
473	50	F	non-GCB	Salvage	CBV	-	Yes	7.1
512	45	M	non-GCB	Salvage	CBV	-	No	46.6
519	44	M	GCB	Consolidation	CBV	-	No	42.5
383	40	F	non-GCB	Consolidation	R-CBV	-	Yes	23.5
419	62	M	non-GCB	Salvage	BEAM	Pneumonia & AF	Yes	18.8
486	49	M	non-GCB	Salvage	CBV	Diarrhea	Yes	6.7
615	55	F	non-GCB	Salvage	CBV	-	Yes	6.0
548	65	M	non-GCB	Salvage	CBV	-	No	32.5
559	32	M	non-GCB	Salvage	CBV	-	No	38.3
601	20	M	GCB	Salvage	CBV	-	No	30.2
617	52	F	non-GCB	Salvage	CBV	-	No	21.8
620	47	M	non-GCB	Salvage	CBV	-	No	17.5

ASCR: autologous stem cell rescue; PFS: progression free survival; M: male; F: female; GCB: germinal center B-cell; CBV: cyclophosphamide, carmustine, etoposide; R-CBV: rituximab, cyclophosphamide, carmustine, etoposide; BEAC: carmustine, etoposide, cytosine arabinoside, cyclophosphamide; BEAM: carmustine, etoposide, cytosine arabinoside, melphalan; AF: atrial fibrillation.

**Table 2:** Primary tumor B cell receptor CDR3 sequences of κ light chain.

Patient No.	Primary Tumor CDR3	TP	LD	Ratio
419	TGTCAGCAAAGTTACAGTATTCCTCGGACGTTC	1	0	86.2%
		2	0	
		3	0	
	TGTCAGCAAAGTTACAGTATTCCTCGGACCTTC	1	1	7.9%
		2	0	
		3	1	
457	CAGCAGTATGGTAGCTCACCGGCGACG	1	1	92.5%
		2	1	
		3	0	
	CAGCAGTATGGTAGCTCACCGGCGACC	1	1	6.4%
		2	1	
		3	1	
548	TGTCAGCAGATTGATACCTGGCCTCGAACCTTC	1	N/A	97.3%
		2		
		3		
	TGTCTGCAGCATAATAGATACCCGCTCACTTTC	1		0.6%
		2		
		3		

Rel. Population: the proportion of one CDR3 clone to the total BCR population. CDR3: the third complementarity determining region; TP: time point; LD: closest Levenshtein distance between the tumor CDR3 sequence and the patient’s peripheral B cell CDR3 repertoire.

## Data Availability

BCR IR sequencing data of the hiGC models are publicly available at the Genome Sequence Archive (GSA) of the National Genomics Data Center (https://ngdc.cncb.ac.cn/, BioProject: PRJCA006296, accessed on 5 April 2021). The link for the reviewer is https://ngdc.cncb.ac.cn/gsa-human/s/82bPTI7Z, accessed on 5 April 2021. To review the RNA-seq data on the GEO database, please visit: https://www.ncbi.nlm.nih.gov/geo/query/acc.cgi?acc=GSE180510, accessed on 5 April 2021, with token “epezeugmvfctfcv”. For other original data, please contact zhu-jun2017@outlook.com.

## Supplementary Materials

The following supporting information can be downloaded at: https://www.mdpi.com/article/10.3390/cancers14194628/s1, Figure S1: Flow cytometry results showing MRC40LB cells expressed CD40L and BAFF; Figure S2: Growth curve of WSU-NHL cultured on the MRC40LB feeder layer or as normal method; Figure S3: Flow cytometry results showing the ratio of mCherry+ cells on days in vitro 14. The ratio labels represented the starting population of mCherry+ cells: Naïve B cells; Figure S4: IgKappa (Igκ) Shannon diversity index (SDI) curves of co-cultured hiGC B and Karpas 422 cells based on the MiXCR and TRUST4 algorithms. Figure S5: Germline V gene usage preference for Heavy chain, Kappa and Lambda light chains; Figure S6: Gini index (GI) and Gini-Simpson Coefficient (GC) of IGH, IGK, and IGL were normalized by the corresponding diversity indices of healthy volunteers and plotted in R. Statistical analysis was conducted with the “rstatix” package in R; Table S1: Top 20 Reads of Karpas 422 cells’ third complementarity determining regions (CDR3) sequences of B cell receptor (BCR) immune repertoire (IR). There was a lack of a dominant BCR clone in the Karpas 422 population; Table S2: Different diversity indices for all peripheral blood samples from patients, either with individual BCR clone or with clusters as established by network analysis.
