# Supplementary material for: The Clonal Diversity of Peripheral B Cell Receptor Immune Repertoire Impaired by Residual Malignant B Cells Predicts Treatment Efficacy in B Cell Lymphoma Patients
Source: Cancers (Basel). 2022 Sep 23;14(19):4628. doi: 10.3390/cancers14194628 (PMC9564088; doi:10.3390/cancers14194628)
Supplement: Supplementary file 1 [file cancers-14-04628-s001.zip › cancers-1844709-supplementary.pdf]

Supplementary Material

1 Supplementary Data

1.1 Construction of the CD40LB Lentiviral Vector

pSLenti-SFH-EGFP-P2A-Puro-CMV-MCS-3xFLAG-WPRE (OBiO Tech Co. Ltd., Shanghai, China) was digested using EcoR I and BamH I. The ORF sequence of the human CD40L [NM_000074.2] and human BAFF [NM_006573.4] were obtained by gene synthesis. A restriction enzyme reaction procedure was performed before gel extraction. The digested gene fragment and vector were integrated to form pSLenti-SFH-EGFP-P2A-Puro-CMV-TNFSF13B-P2A-CD40LG-WPRE, which was then transferred into competent DH5α cells (OBiO Tech Co. Ltd., Shanghai, China). Positive clones were identified by sequencing. Bacteria were incubated in a shaker at 37 ℃ overnight in LB culture medium. Recombinant plasmids were extracted using an EndoFree Maxi Plasmid Kit (TIANGEN, Beijing, China). HEK 293T cells were transfected with pSLenti-SFH-EGFP-P2A-Puro-CMV-TNFSF13B-P2A-CD40LG-WPRE and backbone vectors using Lipofectamine 2000 (Invitrogen, USA). The supernatant was harvested after viral amplification. The acquired lentiviral were stored at -80 ℃.

1.2 Flow cytometry analysis

To detect CD40L and BAFF on MRC40LB cells, the MRC40LB cells were collected by trypsinization, centrifuged at 1000 rpm for 5 min, washed by PBS twice, and then transferred into 1.5 ml EP tubes. The supernatant was discarded after centrifugation. Then MRC40LB cells were re-suspended with 100μl PBS and incubated with anti-CD40L antibody (Biolegend, 310809) or anti-BAFF atibody (Biolegend, 366511) for 30 min in the dark at room temperature.

To assess IgM, IgG, IgE, CD38, CD27, Fas, GL7 expression during a human in vitro germinal center (hiGC) culturing. The hiGC cells on days in vitro (DIV) 0, DIV 8, DIV 12, DIV 19 were stained for 30 min in the dark at room temperature with the following anti-human antibodies as the operation manual described. Group 1 tube was incubated with anti-CD20 antibody (Biolegend, 302322), anti-IgG (Biolegend, 410706), anti-IgM (Biolegend, 314519), anti-IgE (Biolegend, 325513). Group2 tube was incubated with anti-CD20 antibody, anti-CD38 antibody (Biolegend, 356606), anti-CD27 antibody (Biolegend, 356405). Group3 tube was incubated with anti-CD20 antibody, anti-CD95/Fas antibody (Biolegend, 305607), anti-GL7 antibody (Biolegend, 144617). All of these 3 tubes were incubated for 30 min in the dark at room temperature since add antibody.

All flow cytometry (FCM) data were collected on NovoSampler Pro or NovoSampler Q and NovoExpress Software (NovoCyte) and analyzed using NovoExpress Software 1.4.1 (NovoCyte).

1.3 RNA-seq analysis

RNA was extracted from hiGC cells which were cultured on DIV 0, DIV 8, DIV 12, DIV 14, DIV 19 to do RNA-seq analysis by Novogene (Novogene Co., Ltd. Beijing).

In general, RNA was extracted for RNA-sequencing (RNA-seq) using Trizol reagent. Specimens were prepared for RNAseq using TruSeq RNA Library Preparation Kit v2. RNA integrity was verified using the Agilent Bioanalyzer 5400 (Agilent Technologies). Total RNA was used as input material for the RNA sample preparations. Briefly, mRNA was purified from total RNA using poly-T oligo-attached magnetic beads. Fragmentation was carried out using divalent cations under elevated temperature in First Strand Synthesis Reaction Buffer(5X). First strand cDNA was synthesized using random hexamer primer and M-MuLV Reverse Transcriptase, then use RNaseH to degrade the RNA. Second strand cDNA synthesis was subsequently performed using DNA Polymerase I and dNTP. Remaining overhangs were converted into blunt ends via exonuclease/polymerase activities. After adenylation of 3'ends of DNA fragments, adaptor with hairpin loop structure were ligated to prepare for hybridization. In order to select cDNA fragments of preferentially 370~420 bp in length, the library fragments were purified with AMPure XP system (Beckman Coulter, Beverly, USA). Then PCR was performed with Phusion High-Fidelity DNApolymerase, Universal PCR primers and Index (X) Primer. At last, PCR products were purified (AMPure XP system) and library quality was assessed on the Agilent Bioanalyzer 2100 system. The clustering of the index-coded samples was performed on a cBot Cluster Generation System using TruSeq PE Cluster Kit v3-cBot-HS (Illumia) according to the manufacturer's instructions. After cluster generation, the library preparations were sequenced on an Illumina Novaseq platform and 150 bp paired-end reads were generated.

1.4 B cell receptor immune repertoire (BCR IR) sequencing

RNA was extracted from hiGC cells which were cultured on DIV 0, DIV 8, DIV 10, DIV 12, DIV 14, to do BCR IR analysis by GSmed (Gsmed Co., Ltd, Beijing, China).

In general, RNA was extracted for BCR IR analysis using Trizol reagent and the RNA concentration was measured using Qubit RNA HS Assay Kit (Thermo Fisher Scientific, Mas, USA) before storage at -80℃ for future analysis. The integrity and size distribution of total RNA from cells then confirmed using an automated analysis system Agilent 2100 Bioanalyzer with Agilent RNA 6000 Pico Kit (Agilent Technologies, California, USA).

First strand cDNA was synthesized using SuperScript VILO cDNA Synthesis Kit (Thermo Fisher Scientific, Mas, USA) with Oligo (dT)17 primer. The cycling conditions were as follows: 25℃ for 10 min, 42℃ for 60 min and terminate the reaction at 85℃ at 5 min in S1000 Thermal Cycler (Bio-rad, California, USA).

Library preparation: The third complementarity determining regions (CDR3) of BCR were amplified by multiplex PCR. We used the Multi-IR Library Prep Kit that include 267 forward primers annealed to the V region of BCR (IgH, IgK and IgL) and 26 reverse primers annealed to the junction (J) region in order to enrich the maximum possible CDR3. The reaction conditions were as follows: 10 μL 2×KAPA_Enzyme Mix (Roche LifeScience, Swiss), 2 μL BCR Primer Mix, 10 μL cDNA (generally

ranged 200 ng~400 ng) in a 30 μL volume. The cycling conditions were as follows: 95℃ at 4 min for warming up, 30 cycles at 95℃ for 30 sec, 60℃ for 4 min, and finally 72℃ at 5 min for extension. The target amplified fractions (about 100~300 bp) products were kept and purified by Agencourt AMPure XP DNA Clean Beads (Beckman, California, USA) for sequencing. The library presequencing QC confirmed using an automated analysis system Agilent 2100 Bioanalyzer with Agilent DNA 1000 Kit (Agilent Technologies, California, USA).

Sequencing and barcode filtering: Sequencing libraries were prepared using Illumina protocols and sequenced using 150 bp paired-ended sequencing on an Illumina Novaseq 6000 platform (Illumina, California, USA). Using MiXCR (available at http://mixcr.milaboratory.com/ and https://github.com/milaboratory/mixcr/), a universal framework that processes large immune repertoire data from raw sequences to quantitated clonotypes. First, paired- and single-end reads, considering sequence quality, corrects PCR errors were efficiently handled and germline hypermutations were identified. The Killinger algorithm available from GitHub (https://github.com/milaboratory/milib/tree/develop/src/main/java/com/milaboratory/core/alignment) was used to align both reads and aggregates information from both alignments to achieve high V and J gene assignment accuracy in paired-end sequencing analysis.

Then, based on a built-in library of reference germline V, D, J and C gene sequences for human and mouse corresponding to loci from GenBank, MiXCR further assembles identical and homologous reads into clonotypes, correcting for PCR and sequencing errors using a heuristic multilayer clustering. Additionally, it rescues low-quality reads by mapping them to previously assembled high-quality clonotypes to preserve maximal quantitative information. Sequences include incomplete codons and codons that cannot be translated into amino acids (such as the three bases at the beginning of T) will be filtered out through immunarch (available at https://immunarch.com/).

Diversity indices of the hiGC B cells were calculated with a “sub-sampling” strategy, explained in the following scheme and figure:

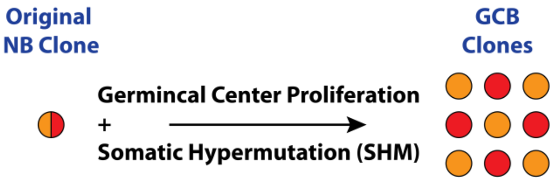

Issue-1: The population of the original NB clone (red-organge circle) was too small for reliable BCR IR sequencing

Solution-1: Start with an adequately large population of NB clones (***green*** dashed sqaure).

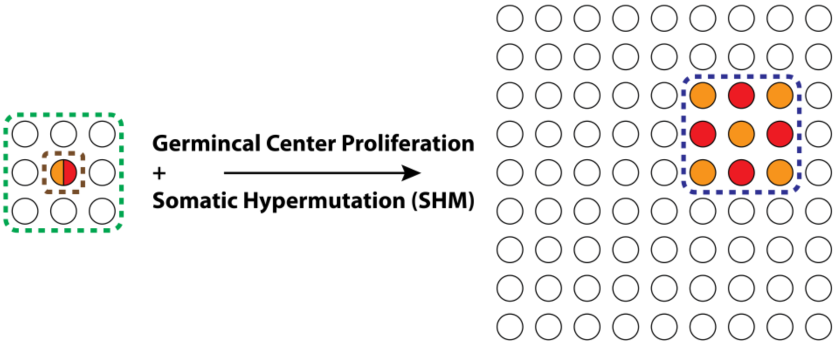

Issue-2: Starting with an adequately large population of NB clones (solution 1), would give rise to a final GCB population too large to be sequenced in its entirety.

Solution-2: Only sequence a fraction of the final GCB population for BCR IR (***blue*** dashed square).

Issue-3: The BCR repertoire of the original NB clones contain clones that were not sampled in the resulting GCB BCR repertoire (white circles).

Solution-3: As we knew the fold of expansion from the original NB clone to the resulting GCB clones (9-fold in the example), we carried out "Sub-sampling" in the BCR IR of the original NB clones (***brown*** dashed square), for the calculation of diversity indices.

For DIV0, 1/2000 of the sequenced BCR IR were sub-sampled.
For DIV8, 1/100 of the sequenced BCR IR were sub-sampled.
For DIV10, 1/10 of the sequenced BCR IR were sub-sampled.
For DIV12 and DIV14, all of the sequenced BCR IR were used without sub-sampling.

The random sub-sampling procedures were iterated for 2500 times. The mean and standard deviation (SD) from the iterated results were used as the diversity indices of each time point.
Our simulation showed that the mean SDI converged on 500 iterations, which supported the use of 2500 iterations for the calculation of SDI.

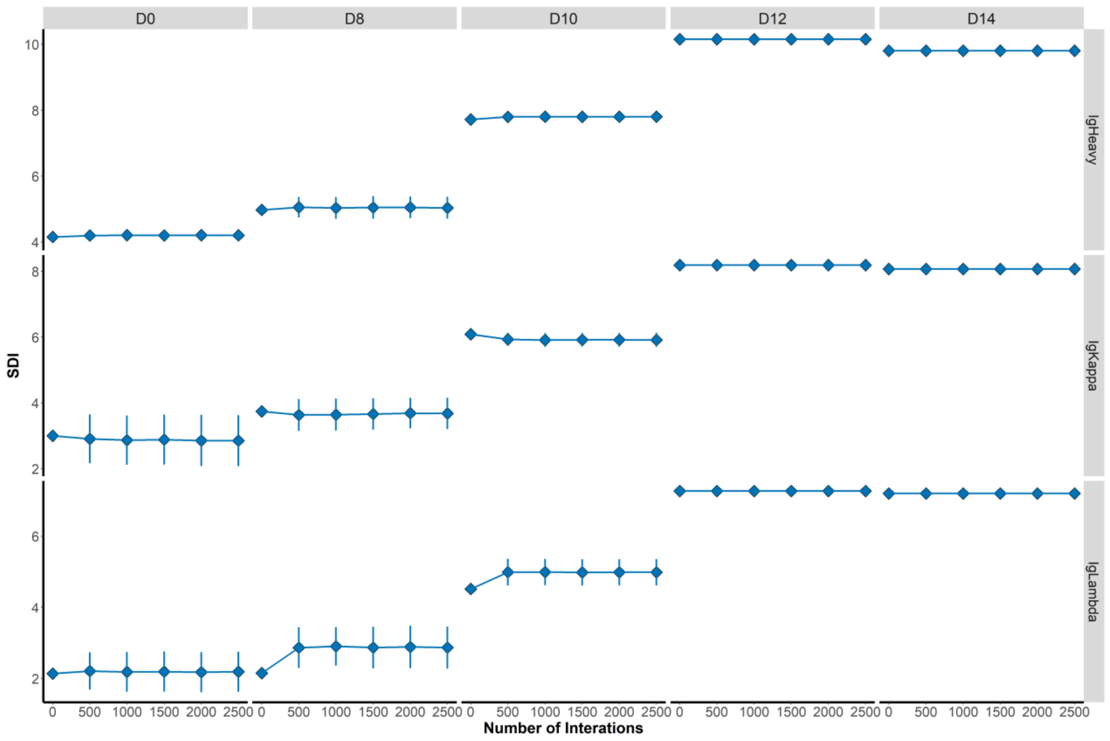
D0
D8
D10
D12
D14
IgHeavy
IgKappa
IgLambda
SDI
Number of Interations
0
500
1000
1500
2000
2500
2
4
6
8
10

2 Supplementary Figures:

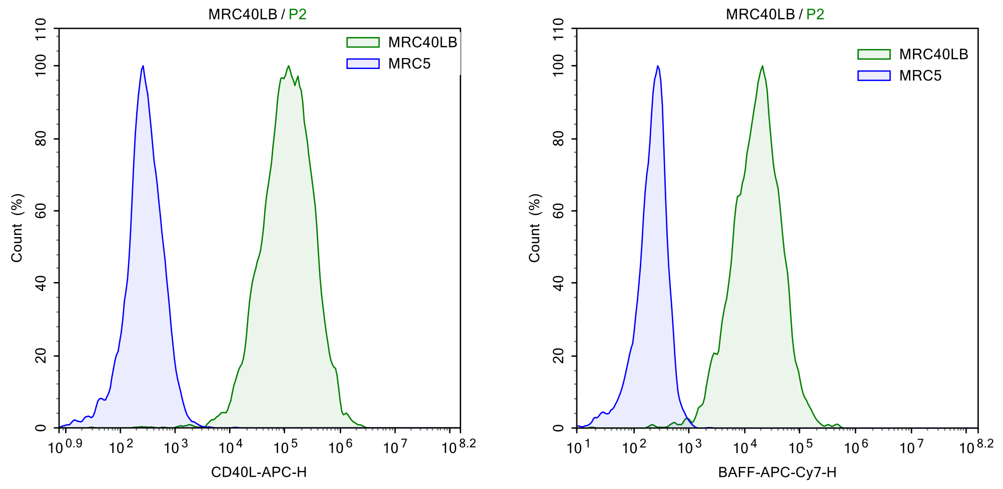

Figure S1 Flow cytometry results showing MRC40LB cells expressed CD40L and BAFF.

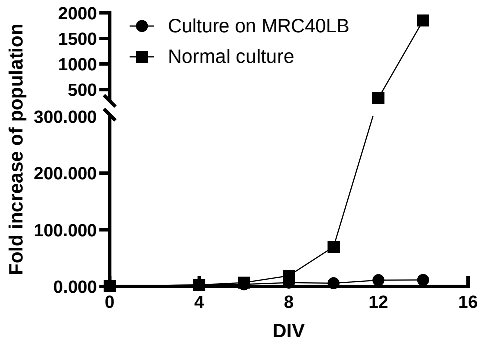

Figure S2 Growth curve of WSU-NHL cultured on the MRC40LB feeder layer or as normal method.

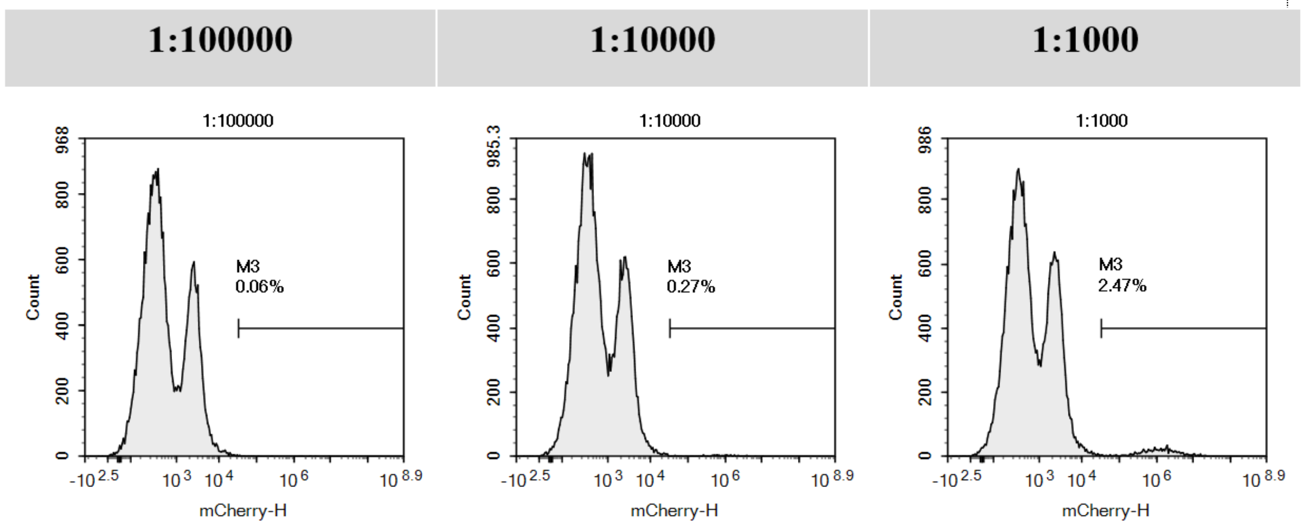

Figure S3 Flow cytometry results showing the ratio of mCherry+ cells on days in vitro 14. The ratio labels represented the starting population of mCherry+ cells: Na ïve B cells.

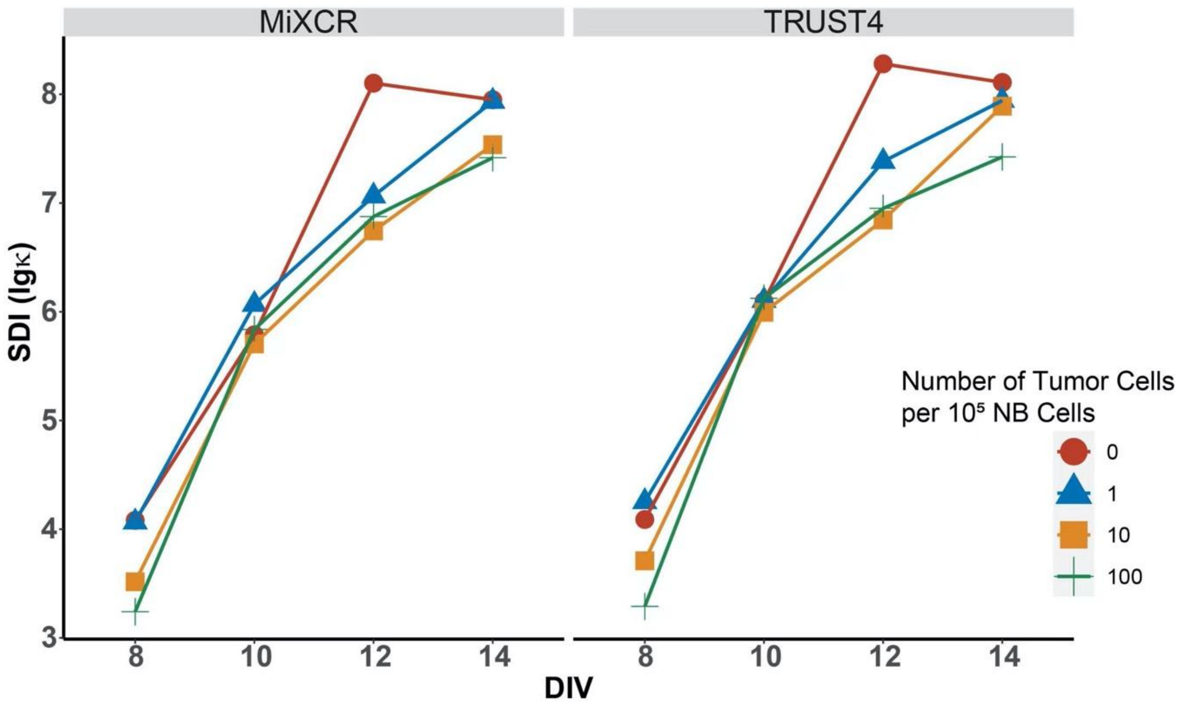

Figure S4 IgKappa (Igκ) Shannon diversity index (SDI) curves of co-cultured hiGC B and Karpas 422 cells based on the MiXCR and TRUST4 algorithms.

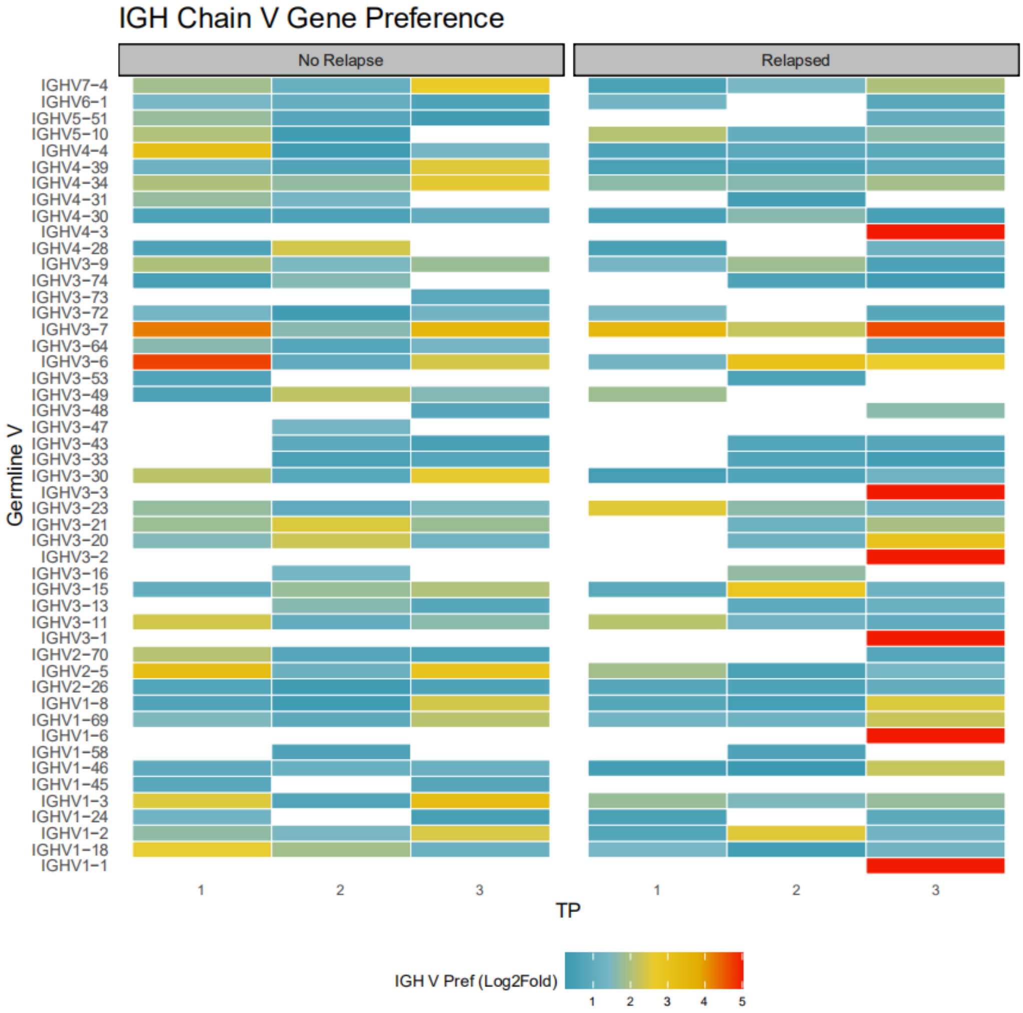
IGH Chain V Gene Preference
No Relapse
Relapsed
IGHV7−4
IGHV6−1
IGHV5−51
IGHV5−10
IGHV4−4
IGHV4−39
IGHV4−34
IGHV4−31
IGHV4−30
IGHV4−3
IGHV4−28
IGHV3−9
IGHV3−74
IGHV3−73
IGHV3−72
IGHV3−7
IGHV3−64
IGHV3−6
IGHV3−53
IGHV3−49
IGHV3−48
IGHV3−47
IGHV3−43
IGHV3−33
IGHV3−30
IGHV3−3
IGHV3−23
IGHV3−21
IGHV3−20
IGHV3−2
IGHV3−16
IGHV3−15
IGHV3−13
IGHV3−11
IGHV3−1
IGHV2−70
IGHV2−5
IGHV2−26
IGHV1−8
IGHV1−69
IGHV1−6
IGHV1−58
IGHV1−46
IGHV1−45
IGHV1−3
IGHV1−24
IGHV1−2
IGHV1−18
IGHV1−1
Germline V
1
2
3
1
2
3
TP
IGH V Pref (Log2Fold)
1
2
3
4
5

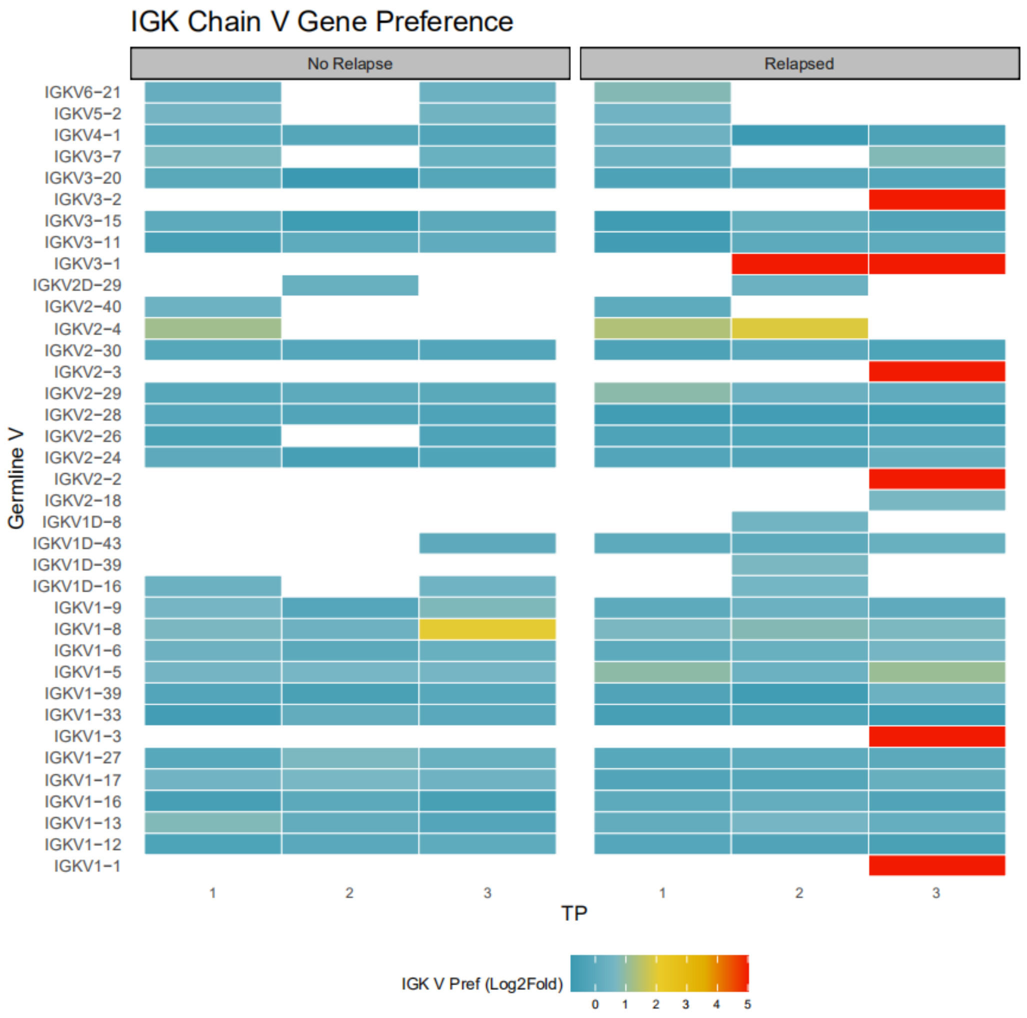
IGK Chain V Gene Preference
No Relapse
Relapsed
Germline V
IGKV6−21
IGKV5−2
IGKV4−1
IGKV3−7
IGKV3−20
IGKV3−2
IGKV3−15
IGKV3−11
IGKV3−1
IGKV2D−29
IGKV2−40
IGKV2−4
IGKV2−30
IGKV2−3
IGKV2−29
IGKV2−28
IGKV2−26
IGKV2−24
IGKV2−2
IGKV2−18
IGKV1D−8
IGKV1D−43
IGKV1D−39
IGKV1D−16
IGKV1−9
IGKV1−8
IGKV1−6
IGKV1−5
IGKV1−39
IGKV1−33
IGKV1−3
IGKV1−27
IGKV1−17
IGKV1−16
IGKV1−13
IGKV1−12
IGKV1−1
1
2
3
1
2
3
TP
IGK V Pref (Log2Fold)
0
1
2
3
4
5

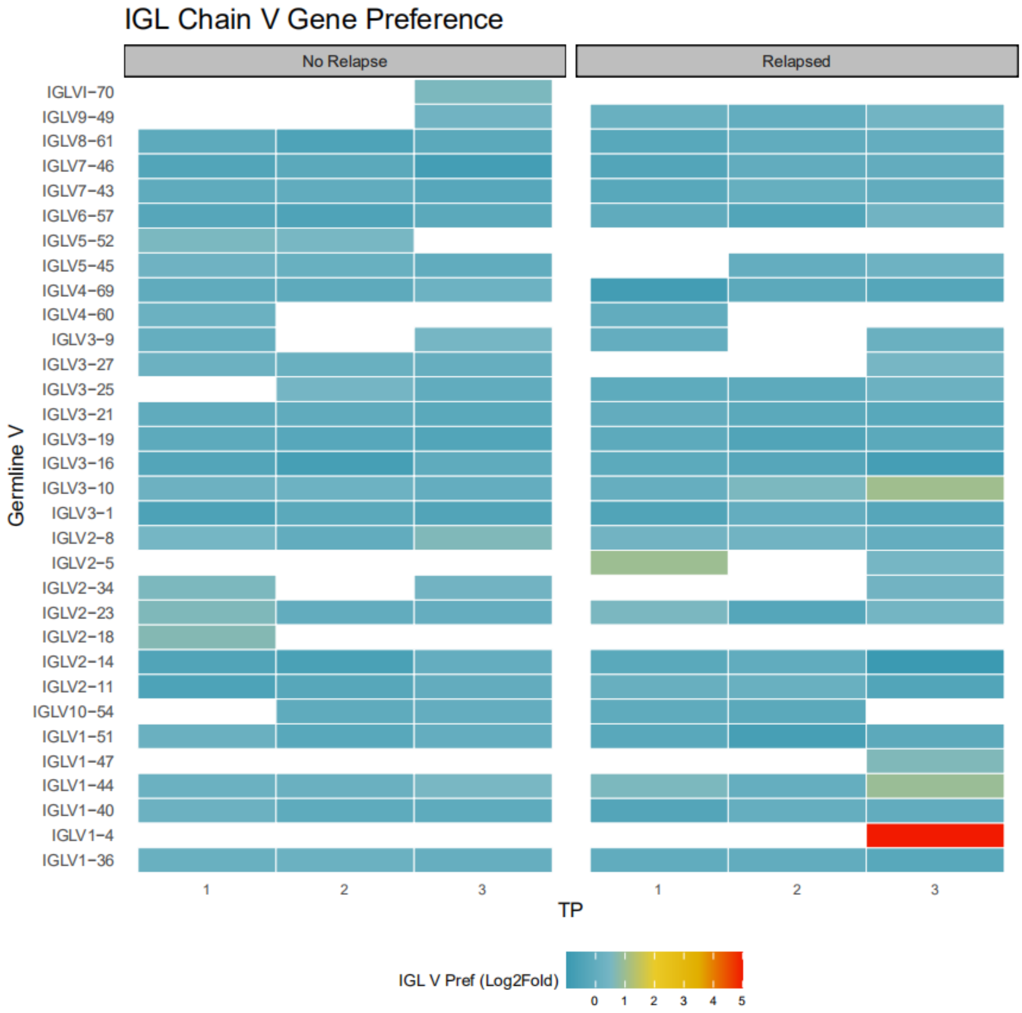

Figure S5 Germline V gene usage preference for Heavy chain, Kappa and Lambda light chains.

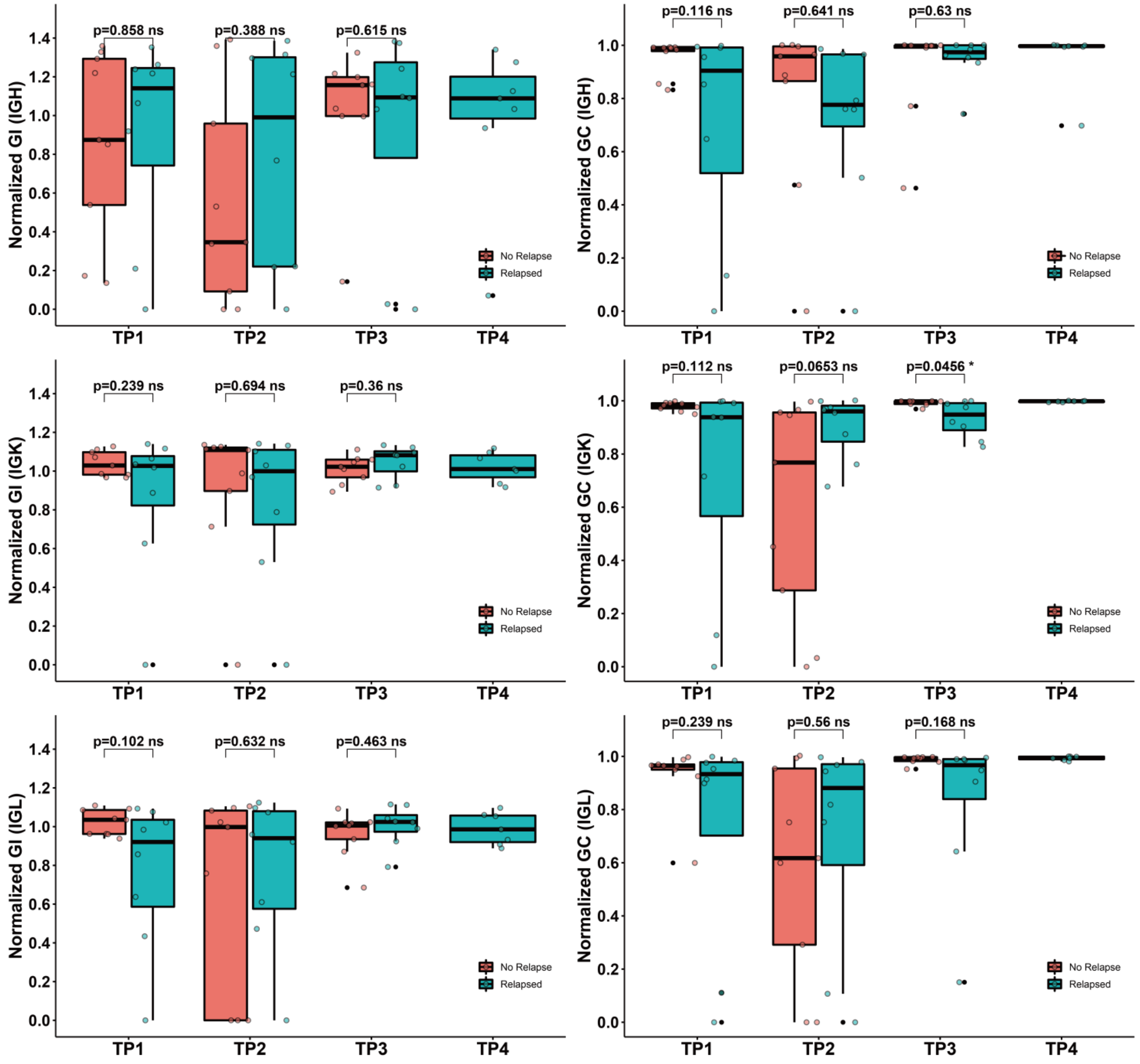

Figure S6 Gini index (GI) and Gini-Simpson Coefficient (GC) of IGH, IGK and IGL were normalized by the corresponding diversity indices of healthy volunteers and plotted in R. Statistical analysis was conducted with the “rstatix” package in R.

3 Supplementary Tables:

Table S1: Top 20 Reads of Karpas 422 cells' the third complementarity determining regions (CDR3) sequences of B cell receptor (BCR) immune repertoire (IR). There was a lack of a dominant BCR clone in the Karpas 422 population

Rank	**IgHeavy**	
	CDR3	Reads
1	TGTGCGAGAGATATTCCATCGTCTAGCAGCGACTGGGTTTCCCTACAGTTTGACTACTGG	605
2	TGTACTAGACATTCTTTATCCGAGGGCGACTACGGTGACTACCGCTACTGGTACTTCGATCTCTGG	511
3	TGTGCGAGACGCGTCCCGAGGCCTACCTATTGTAGTAATAATACCTGCTCTTACTGGTACTTCGATCTCTGG	411
4	TGTACTAGTCTTCAGTATTACTATGATAGTAGTGGTTATTACTTAGATTACTACTACTACTACATGGACGTCTGG	329
5	TGTGCGAGACATCCGAATGACATTCACTCTTACTTCGATCTCTGG	249
6	TGTGCGAGACGCGTTCCGAGGCCTACCTATTGTAGTAATACCAGCTGCTCTTACTGGTACTTCGATCTCTGG	238
7	TGTGCGAGAGCCAGGGTTTACTATGATAGTAGTGGTCCCAATGCGGAGGACTGGTACTTCGATCTCTGG	207
8	TGTGCGAGAATGAGCCATTACTATGATAGTAGTGGTTATTTCGATCTCTGG	177
9	TGTACTAGACGAGGCAGTAACTACTTTGACTACTGG	160
10	TGTGCGAGGGTCCCCCTCTTGGGGGGCTGGGAGTACTGGTACTTCGATCTCTGG	157
11	TGTGCCACTACGATTTTTGGAGTGGTTATTATTTCCGATCTAACTGGGTACTTCGATCTCTGG	153
12	TGTACCACAGCCGGGACGTATTACTATGACAGTAGTGGTTACCGGTACTTCGATCTCTGG	143
13	TGTGCGAGAGATGGAATGGAGAGCAGCAGCCACTACGACCTCCTCTACTGGTACTTCGATCTCTGG	134
14	TGTGCGAGAGGGGTTTATTACTATGGTAGTAGTGGTTATGGACCCTCCTGGTACTTCGATCTCTGG	132
15	TGTCTCGTCTATTATGGTAGTAGTGTTTCTGCCTACTGG	127
16	TGTGCACGGATACTTGAGGGTGGGAGCTACTACTTTGATGCTTTTGATATCTGG	111
17	TGTGCGAGAGGGGGAACTGGGGATAACGCTTACTGGTACTTCGATCTCTGG	108
18	TGTGCACACACAGCTTTCTATGGTTCGGGGAGTTATCAAGTCTGGTACTTCGATCTCTGG	107
19	TGTGCGAGAGAGTTTCTCGCTATAGCAGCAGAGGGGTATTACTATGATAGTTTGGGTGCTCTTGATATCTGG	104
20	TGTGCGAGAGCTCTTACGATTTTTGGAGTGGCCCCTTTTGACTACTGG	97

	**IgKappa**	**IgLambda**

Rank	CDR3	Reads	CDR3	Reads
1	TGTCAACAGTATGATAATCTCCCGCTCACTTTC	8943	TGCAGCTCATATACAAGCAGCAGCACTTGGGTGTTC	5973
2	TGTCAACAGTATGATAATCTCCCTCTCACTTTC	4341	TGTATGATTTGGCACAGCAGCGCTTGGGTGTTC	4932
3	TGCCAACAGTATAATAGTTACCCTCTCACTTTC	4115	TGTAACTCCCGGGACAGCAGTGGTAACCATGTGGTATTC	4832
4	TGTCAGCAATATTATAGTACTCCGCTCACTTTC	3907	TGTGTGCTGTATATGGGTAGTGGCATTTGGGTGTTC	4593
5	TGTCAGCAATATTATAGTACTCCTCTCACTTTC	2981	TGTCAGGCGTGGGACAGCAGCACTGTGGTATTC	4133
6	TGTCAACAGTATGATAATCTCCCGATCACCTTC	2587	TGCAGCTCATATACAAGCAGCAGCACTTATGTCTTC	3426
7	TGTCAGCAGTATGGTAGCTCACCGCTCACTTTC	2487	TGTCAGTCTTATGATAGCAGCAATTGGGTGTTC	3290
8	TGTCAGCAGTATGGTAGCTCACTCACTTTC	2174	TGTAACTCCCGGGACAGCAGTGGTAACCATTGGGTGTTC	3211
9	TGTCAACAGTATGATAATCTCCCGCTCACCTTC	2086	TGTCAATCAGCAGACAGCAGTGGTACTTATGTGGTATTC	2563
10	TGCATGCAAGCTCTACAAACTCCTCTCACTTTC	1792	TGCAGCTCATATACAAGCAGCAGCACTTTGGTATTC	2540
11	TGTCAGCAATATTATAGTACTCCGTACACTTTC	1669	TGTGGGGCAGACCATGGCAGTGGGAGCAACTTCGTGTGGGTGTTC	2385
12	TGTCAACAGTATGATAATCTCCCGTACACTTTC	1658	TGTCAGACCTGGGGCACTGGCATTCAGGTGTTC	2195
13	TGTCAGCAGTATAATAACTGGCCTCCGCTCACTTTC	1615	TGTCAGTCTTATGATAGCAGCAATCATTGGGTGTTC	1977
14	TGCATGCAAGCTCTACAAACTCCGCTCACTTTC	1612	TGCTGCTCATATGCAGGTAGTAGCACTTGGGTGTTC	1908
15	TGTCAACAGCATGATAATCTCCCGCTCACTTTC	1554	TGTATGATTTGGCCAAGCAATGCTTGGGTGTTC	1888
16	TGTCAACAGAGTTACAGTACCCCGCTCACTTTC	1548	TGTCAGGTGTGGGATAGTAGTAGTGATCATGTGGTATTC	1799
17	TGCATGCAAGCTCTACAAACTCCGTACACTTTC	1499	TGTAACTCCCGGGACAGCAGTGGTAACCATCTGGTATTC	1781
18	TGTCAACAGAGTTACAGTACCCCTCTCACTTTC	1463	TGTATGATTTGGCACAGCAGCGCTGTGGTATTC	1676
19	TGTCAACAGTATGATAATCTCCCCCTCACTTTC	1455	TGTCAGGTGTGGGATAGTAGTAGTGATCATTGGGTGTTC	1607
20	TGTCAACAGAGTTACAGTACCCCGATCACCTTC	1380	TGTGCAGCATGGGATGACAGCCTGAATGGTTGGGTGTTC	1327

CDR3: the third complementarity determining regions.

Table S2: Different diversity indices for all peripheral blood samples from patients, either with individual BCR clone or with clusters as established by network analysis

(Some patients' samples before the administration of the conditioning regimen (TP1) and immediately before stem cell transfusion (TP2) contained B cell receptor (BCR) clones too few to be reliably sequenced, producing indices of 0)

PID	IGType	TP	Gini Index	Gini Index (cluster)	Gini-Simpson Coeff.	Gini-Simpson Coeff. (cluster)	Shannon Index	Shannon Index (cluster)
383	IgHeavy	1	0.381	0.380	0.647	0.889	1.419	1.392
383	IgHeavy	2	0.078	0.078	0.967	0.929	2.342	2.342
383	IgHeavy	3	0.000	0.000	1.000	0.916	2.197	2.197
383	IgHeavy	4	0.370	0.347	0.993	0.998	5.567	5.525
383	IgKappa	1	0.451	0.447	0.715	0.856	2.215	2.011
383	IgKappa	2	0.344	0.349	0.873	0.954	2.696	2.365
383	IgKappa	3	0.472	0.462	0.902	0.921	3.006	2.782
383	IgKappa	4	0.400	0.424	0.997	0.999	6.961	5.590
383	IgLambda	1	0.379	0.355	0.911	0.967	3.106	3.058
383	IgLambda	2	0.208	0.171	0.941	0.961	2.846	2.579
383	IgLambda	3	0.452	0.441	0.946	0.969	3.330	3.325
383	IgLambda	4	0.392	0.399	0.997	0.999	6.576	6.048
419	IgHeavy	1	0.444	0.442	0.852	0.964	2.266	2.234
419	IgHeavy	2	0.079	0.099	0.964	0.949	2.843	2.730
419	IgHeavy	3	0.370	0.357	0.962	0.991	3.818	3.784
419	IgHeavy	4	0.403	0.403	0.697	0.969	2.058	2.050
419	IgKappa	1	0.387	0.401	0.936	0.993	3.507	3.150
419	IgKappa	2	0.231	0.263	0.975	0.995	4.735	4.433
419	IgKappa	3	0.399	0.411	0.988	0.998	5.588	5.008
419	IgKappa	4	0.441	0.447	0.997	0.999	6.535	5.711
419	IgLambda	1	0.281	0.295	0.897	0.984	3.301	3.173
419	IgLambda	2	0.270	0.271	0.816	0.989	3.232	3.223
419	IgLambda	3	0.350	0.350	0.987	0.997	5.155	4.951
419	IgLambda	4	0.412	0.418	0.996	0.999	6.137	5.747
457	IgHeavy	1	0.436	0.445	0.997	1.000	6.828	5.894
457	IgHeavy	2	0.464	0.487	0.985	0.999	5.322	4.114
457	IgHeavy	3	0.393	0.401	0.999	1.000	8.794	7.989
457	IgHeavy	4	0.335	0.320	1.000	1.000	9.811	9.279
457	IgKappa	1	0.464	0.486	0.995	0.993	6.256	3.452
457	IgKappa	2	0.481	0.477	0.964	0.970	3.951	3.059
457	IgKappa	3	0.446	0.489	0.998	0.998	7.445	3.255
457	IgKappa	4	0.407	0.467	0.999	1.000	8.248	4.302
457	IgLambda	1	0.451	0.457	0.982	0.990	4.931	3.533
457	IgLambda	2	0.474	0.483	0.966	0.985	4.099	3.017
457	IgLambda	3	0.437	0.473	0.993	0.998	6.306	3.814

457	IgLambda	4	0.401	0.447	0.997	0.999	7.353	4.603
473	IgHeavy	1	0.000	0.000	0.000	0.286	0.000	0.000
473	IgHeavy	2	0.471	0.488	0.758	0.838	2.445	1.292
473	IgHeavy	3	0.444	0.466	0.983	0.999	6.214	4.848
473	IgHeavy	4	0.390	0.409	0.996	1.000	8.221	7.383
473	IgKappa	1	0.000	0.000	0.000	0.286	0.000	0.000
473	IgKappa	2	0.493	0.496	0.760	0.811	2.167	1.443
473	IgKappa	3	0.471	0.464	0.974	0.993	5.223	4.153
473	IgKappa	4	0.437	0.474	0.998	0.999	7.434	4.438
473	IgLambda	1	0.000	0.000	0.000	0.286	0.000	0.000
473	IgLambda	2	0.484	0.493	0.751	0.640	2.069	0.762
473	IgLambda	3	0.453	0.447	0.988	0.998	5.528	4.558
473	IgLambda	4	0.435	0.466	0.990	0.999	6.433	4.364
486	IgHeavy	1	0.329	0.319	0.993	0.997	5.488	5.400
486	IgHeavy	2	0.434	0.434	0.501	0.750	1.114	1.046
486	IgHeavy	3	0.390	0.376	0.933	0.987	3.412	3.351
486	IgKappa	1	0.444	0.476	0.997	1.000	7.409	4.546
486	IgKappa	2	0.449	0.443	0.954	0.958	4.139	3.770
486	IgKappa	3	0.403	0.441	0.997	0.996	6.663	4.520
486	IgLambda	1	0.435	0.458	0.996	1.000	6.960	5.424
486	IgLambda	2	0.407	0.404	0.977	0.977	4.156	3.984
486	IgLambda	3	0.408	0.416	0.984	0.997	5.437	4.674
512	IgHeavy	1	0.463	0.483	0.977	0.998	5.015	3.845
512	IgHeavy	2	0.000	0.000	0.000	0.286	0.000	0.000
512	IgHeavy	3	0.371	0.377	0.999	1.000	8.723	8.020
512	IgKappa	1	0.479	0.489	0.970	0.978	4.619	2.944
512	IgKappa	2	0.000	0.000	0.000	0.286	0.000	0.000
512	IgKappa	3	0.446	0.491	0.997	0.998	7.308	2.900
512	IgLambda	1	0.482	0.490	0.923	0.962	3.525	2.556
512	IgLambda	2	0.000	0.000	0.000	0.286	0.000	0.000
512	IgLambda	3	0.443	0.477	0.994	0.999	6.416	4.004
519	IgHeavy	1	0.437	0.452	0.989	0.999	6.473	5.309
519	IgHeavy	2	0.000	0.000	1.000	0.962	3.367	3.367
519	IgHeavy	3	0.429	0.441	0.998	1.000	7.179	6.111
519	IgKappa	1	0.467	0.466	0.981	0.998	5.679	4.582
519	IgKappa	2	0.495	0.498	0.450	0.745	1.326	0.493
519	IgKappa	3	0.463	0.480	0.984	0.997	6.327	3.957
519	IgLambda	1	0.460	0.467	0.985	0.997	5.421	4.139
519	IgLambda	2	0.000	0.000	1.000	0.921	2.303	2.303
519	IgLambda	3	0.451	0.455	0.991	0.998	5.672	4.394
535	IgHeavy	1	0.452	0.000	0.133	0.004	0.440	0.000
535	IgHeavy	2	0.000	0.000	0.000	0.667	0.000	0.000
535	IgHeavy	3	0.495	0.462	0.741	0.703	2.030	1.482
535	IgHeavy	4	0.457	0.376	0.998	0.999	7.135	6.526
535	IgKappa	1	0.497	0.400	0.118	0.000	0.450	0.000

535	IgKappa	2	0.000	0.000	1.000	0.890	1.386	1.386
535	IgKappa	3	0.489	0.217	0.845	0.832	2.281	1.991
535	IgKappa	4	0.478	0.460	0.996	0.922	6.352	3.289
535	IgLambda	1	0.475	0.250	0.111	0.004	0.410	0.006
535	IgLambda	2	0.000	0.000	0.000	0.286	0.000	0.000
535	IgLambda	3	0.492	0.418	0.150	0.079	0.519	0.216
535	IgLambda	4	0.468	0.408	0.991	0.977	5.248	3.965
548	IgHeavy	1	0.193	0.193	0.854	0.949	2.383	2.383
548	IgHeavy	2	0.121	0.121	0.957	0.983	3.868	3.868
548	IgHeavy	3	0.414	0.402	0.997	1.000	7.057	7.002
548	IgKappa	1	0.430	0.418	0.990	0.995	5.219	4.884
548	IgKappa	2	0.486	0.485	0.767	0.925	2.296	2.158
548	IgKappa	3	0.456	0.465	0.968	0.992	5.942	4.687
548	IgLambda	1	0.425	0.421	0.967	0.984	4.318	4.277
548	IgLambda	2	0.478	0.478	0.598	0.955	1.871	1.848
548	IgLambda	3	0.449	0.447	0.990	0.997	5.710	5.042
559	IgHeavy	1	0.313	0.319	0.831	0.970	2.465	2.412
559	IgHeavy	2	0.343	0.324	0.999	1.000	7.656	7.598
559	IgHeavy	3	0.051	0.054	0.998	0.987	4.563	4.551
559	IgKappa	1	0.421	0.432	0.949	0.995	3.742	3.386
559	IgKappa	2	0.431	0.454	0.995	0.999	6.477	4.811
559	IgKappa	3	0.422	0.465	0.997	0.998	6.970	4.394
559	IgLambda	1	0.457	0.458	0.598	0.970	1.855	1.828
559	IgLambda	2	0.441	0.442	0.991	0.999	5.601	4.929
559	IgLambda	3	0.413	0.439	0.995	0.999	6.216	4.870
592	IgHeavy	1	0.484	0.377	0.990	0.989	5.361	4.815
592	IgHeavy	2	0.496	0.446	0.791	0.749	2.217	1.542
592	IgHeavy	3	0.492	0.442	0.952	0.936	3.723	3.070
592	IgHeavy	4	0.480	0.421	0.993	0.997	6.137	5.579
592	IgKappa	1	0.487	0.356	0.991	0.987	5.138	4.597
592	IgKappa	2	0.498	0.400	0.677	0.641	1.570	1.180
592	IgKappa	3	0.494	0.427	0.919	0.912	3.483	3.083
592	IgKappa	4	0.487	0.467	0.993	0.895	5.692	3.073
592	IgLambda	1	0.483	0.360	0.974	0.971	4.222	3.781
592	IgLambda	2	0.496	0.250	0.107	0.000	0.415	0.000
592	IgLambda	3	0.491	0.461	0.903	0.764	2.845	1.857
592	IgLambda	4	0.484	0.431	0.978	0.951	4.653	3.529
598	IgHeavy	1	0.486	0.434	0.989	0.989	5.305	4.747
598	IgHeavy	2	0.498	0.488	0.474	0.329	1.348	0.510
598	IgHeavy	3	0.474	0.396	0.990	0.992	5.323	4.707
598	IgKappa	1	0.491	0.463	0.987	0.904	5.049	2.972
598	IgKappa	2	0.483	0.000	0.033	0.000	0.136	0.000
598	IgKappa	3	0.484	0.434	0.991	0.938	5.264	3.357
598	IgLambda	1	0.490	0.470	0.962	0.805	3.981	2.225

598	IgLambda	2	0.000	0.000	0.000	0.286	0.000	0.000
598	IgLambda	3	0.483	0.420	0.950	0.851	3.645	2.542
601	IgHeavy	1	0.049	0.061	0.991	0.956	3.142	3.090
601	IgHeavy	2	0.033	0.033	0.995	0.959	3.233	3.233
601	IgHeavy	3	0.356	0.336	0.997	0.999	6.174	6.118
601	IgKappa	1	0.449	0.437	0.958	0.978	3.740	3.564
601	IgKappa	2	0.489	0.490	0.287	0.728	0.856	0.579
601	IgKappa	3	0.441	0.465	0.997	0.998	7.003	4.968
601	IgLambda	1	0.424	0.418	0.961	0.979	3.526	3.488
601	IgLambda	2	0.488	0.488	0.616	0.745	1.170	1.169
601	IgLambda	3	0.444	0.445	0.994	0.999	6.334	5.514
602	IgHeavy	1	0.475	0.398	0.992	0.995	5.524	4.987
602	IgHeavy	2	0.486	0.391	0.864	0.841	2.605	2.025
602	IgHeavy	3	0.415	0.326	0.999	1.000	7.512	7.076
602	IgKappa	1	0.485	0.438	0.991	0.948	5.417	3.516
602	IgKappa	2	0.491	0.391	0.945	0.922	3.471	2.804
602	IgKappa	3	0.462	0.456	0.997	0.945	6.587	3.444
602	IgLambda	1	0.480	0.412	0.964	0.927	3.862	3.063
602	IgLambda	2	0.484	0.435	0.291	0.200	0.870	0.425
602	IgLambda	3	0.452	0.405	0.977	0.972	4.947	3.739
615	IgHeavy	1	0.075	0.075	0.955	0.924	2.210	2.210
615	IgHeavy	2	0.275	0.275	0.760	0.915	1.610	1.610
615	IgHeavy	3	0.010	0.010	0.999	0.976	3.905	3.905
615	IgHeavy	4	0.025	0.025	0.998	0.978	3.951	3.951
615	IgKappa	1	0.273	0.292	0.938	0.996	4.360	4.215
615	IgKappa	2	0.423	0.471	0.997	1.000	7.436	4.778
615	IgKappa	3	0.478	0.480	0.826	0.995	2.690	2.505
615	IgKappa	4	0.465	0.486	0.993	0.999	6.911	4.520
615	IgLambda	1	0.192	0.204	0.950	0.992	4.273	4.193
615	IgLambda	2	0.424	0.442	0.994	0.999	6.510	5.380
615	IgLambda	3	0.460	0.463	0.641	0.988	2.070	1.909
615	IgLambda	4	0.465	0.478	0.983	0.999	6.012	4.692
617	IgHeavy	1	0.062	0.062	0.990	0.965	3.376	3.376
617	IgHeavy	2	0.124	0.136	0.964	0.966	3.252	3.141
617	IgHeavy	3	0.435	0.435	0.462	0.966	1.447	1.447
617	IgKappa	1	0.428	0.421	0.975	0.985	4.077	3.816
617	IgKappa	2	0.311	0.333	0.955	0.994	4.256	3.987
617	IgKappa	3	0.405	0.394	0.987	0.997	4.966	4.583
617	IgLambda	1	0.425	0.422	0.948	0.973	3.386	3.359
617	IgLambda	2	0.335	0.341	0.952	0.993	3.945	3.873
617	IgLambda	3	0.385	0.378	0.980	0.995	4.447	4.285
620	IgHeavy	1	0.305	0.299	0.982	0.993	4.493	4.380
620	IgHeavy	2	0.190	0.190	0.886	0.952	2.513	2.513
620	IgHeavy	3	0.357	0.348	0.770	0.929	2.097	1.986
620	IgKappa	1	0.422	0.453	0.997	0.999	6.877	4.927

620	IgKappa	2	0.391	0.358	0.965	0.989	4.588	4.250
620	IgKappa	3	0.390	0.365	0.997	0.999	6.498	5.678
620	IgLambda	1	0.414	0.426	0.995	0.999	6.160	5.326
620	IgLambda	2	0.452	0.433	0.750	0.890	2.463	2.436
620	IgLambda	3	0.303	0.277	0.995	0.998	5.866	5.573

PID: patients' ID; TP: time point; TP1: before the administration of the conditioning regimen; TP2: immediately before stem cell transfusion; TP3: 6-8 weeks after high-dose therapy and autologous stem cell rescue; TP4: the time point of confirmed relapse after autologous stem cell rescue.
